# Distinct Omicron longitudinal memory T cell profile and T cell receptor repertoire associated with COVID-19 hospitalisation

**DOI:** 10.3389/fimmu.2025.1549570

**Published:** 2025-04-02

**Authors:** Gavin Markey, Joseph McLaughlin, Darren McDaid, Seodhna M. Lynch, Andrew English, H. Denis Alexander, Martin Kelly, Manav Bhavsar, Victoria McGilligan, Shu-Dong Zhang, Elaine K. Murray, Taranjit Singh Rai, Colum Walsh, Anthony J. Bjourson, Priyank Shukla, David S. Gibson

**Affiliations:** ^1^ Personalised Medicine Centre, School of Medicine, Ulster University, Londonderry, United Kingdom; ^2^ Medical Directorate, Clinical and Translational Research and Innovation Centre, Londonderry, United Kingdom; ^3^ School of Health and Life Sciences, Teesside University, Middlesborough, United Kingdom; ^4^ Intensive Care Unit, Western Health Social Care Trust, Londonderry, United Kingdom; ^5^ Genomic Medicine Research Group, School of Biomedical Science, Ulster University, Coleraine, United Kingdom; ^6^ Biomedical and Clinical Sciences Division, Department for Cell and Neurobiology, Faculty of Medicine, Linköping University, Linköping, Sweden

**Keywords:** SARS-CoV-2, COVID-19, T cell immunity, T cell receptor (TCR) recognition, antigen presentation, adaptive immunity, immuno-informatics

## Abstract

SARS-CoV-2 has claimed more than 7 million lives worldwide and has been associated with prolonged inflammation, immune dysregulation and persistence of symptoms following severe infection. Understanding the T cell mediated immune response and factors impacting development and continuity of SARS-CoV-2 specific memory T cells is pivotal for developing better therapeutic and monitoring strategies for those most at risk from COVID-19. Here we present a comprehensive analysis of memory T cells in a convalescent cohort (n=20), three months post Omicron infection. Utilising flow cytometry to investigate CD4^+^CD45RO^+^ and CD8^+^CD45RO^+^ memory T cell IL-2 expression following Omicron (B.1.1.529/BA.1) peptide pool stimulation, alongside T cell receptor repertoire profiling and RNA-Seq analysis, we have identified several immunological features associated with hospitalised status. We observed that while there was no significant difference in median CD4^+^CD45RO^+^ IL-2^+^ and CD8^+^ CD45RO^+^ IL-2^+^ memory T cell count between subgroups, the hospitalised subgroup expressed significantly more IL-2 per cell following Omicron peptide pool exposure in the CD8^+^CD45RO^+^ population (p <0.03) and trended towards significance in CD4^+^CD45RO^+^ cells (p <0.06). T cell receptor repertoire analysis found that the non-hospitalised subgroup had a much higher number of circulating clonotypes, targeting a wider range of predominantly MHC-I epitopes across the SARS-CoV-2 genome. Several immunodominant epitopes, conserved between both subgroups, were observed, however hospitalised individuals were less likely to express putative HLA alleles responsible for pMHC presentation which may impact TCR affinity. We observed a bias towards shorter CDR3 segments in TCRβ repertoire analysis within the hospitalised subgroup, alongside lower rates of repertoire overlap in CDR3 sequences compared to the non-hospitalised subgroup. We found a significant proportion of TCRs targeted epitopes along the SARS-CoV-2 genome including non-structural proteins, responsible for viral replication and immune evasion. These findings highlight how the continuity of T cell based protective immunity is impacted by both the viral replication cycle of SARS-CoV-2 upon intracellular and innate immune responses, and HLA-type upon TCR affinity and clonotype formation. Our novel Epitope Target Analysis Pipeline (Epi-TAP) could prove beneficial in development of new therapeutic strategies through rapid identification of shared immunodominant epitopes across non-hospitalised and hospitalised subgroups.

## Introduction

COVID-19 is a highly infectious and potentially fatal disease that has claimed more than 7 million lives worldwide over the past five years (1). In severe cases, COVID-19 is known to cause prolonged viral pneumonia, acute respiratory distress syndrome (ARDS), and potentially fatal multi-organ failure. Severity of infection is often linked to dysregulation of immune responses following activation of NF-κB and MAPK signalling pathways. SARS-CoV-2 viral proteins have been shown to disrupt type I and III interferon (IFN) production, cleaving transcription factor IRF3 and inhibiting mRNA transcription. During infection activation of inflammasome sensor NLRP3 leads to cleavage of gasdermin D (GSDMD) induing pyroptosis, releasing IL-1β and IL-18 leading to further cytokine production. Elevated levels of IL-1β and IL-18 correlate with disease severity and poorer clinical outcomes (2, 3).

In the early stages of infection macrophages, dendritic cells and natural killer (NK) cells are recruited to the site of infection, inducing the production of pro-inflammatory cytokines and chemokines that attract monocytes, macrophages and T cells. Recruited CD4^+^ and CD8^+^ T cells in turn produce pro-inflammatory proteins, resulting in the recruitment and proliferation of various T helper (Th) and cytotoxic T cells (CTLs) leading to the production of antibodies by B cells working in synergy with Th cells, and memory T cells that target and eliminate the virus should reinfection occur (4). Although several studies have reported on the duration, quality and factors influencing T cell based protective immunity to SARS-CoV-2, there is limited longitudinal evidence of these features association with Omicron (B.1.1.529) acute phase infection severity and hospitalisation. We therefore investigated if differences existed in IL-2 expression as a key cytokine promoting memory T cell persistence, CD158b expression as a key modulator of immune response, and T cell receptor repertoire in hospitalised and non-hospitalised cases.Interleukin-2 (IL-2) is a critical cytokine primarily produced by activated T cells, particularly CD4+ T-helper cells. It plays a central role in T cell proliferation, differentiation, and function, essential for a strong immune response (5). IL-2 promotes the expansion, survival, and effectiveness of T cells after they recognise an antigen, ensuring a sustained immune response (6). IL-2 is elevated along with other pro-inflammatory cytokines in severe COVID-19 (7). Rapid IL-2 production following T cell activation serves as a key marker for evaluating immune responses, particularly during infections such as SARS-CoV-2. When T cells encounter SARS-CoV-2, they secrete IL-2, triggering the activation and proliferation of other immune cells. This early production of IL-2 is crucial for assessing the magnitude and quality of the immune response, providing insights into T cell based immune memory (8, 9). IL-2 levels can indicate the presence of memory T cells, which are vital for quick and effective responses to future infections (9). Elevated IL-2 production is crucial for promoting the proliferation and persistence of memory T cells, enhancing the body’s capacity to manage persistent infection or inflammation (10, 11). Furthermore, assessing IL-2 production is useful for evaluating polyfunctional T cells following vaccination and natural exposure to SARS-CoV-2 (12, 13).

CD158b, also known as KIR2DL2/L3/S2, is a receptor typically associated with natural killer (NK) cells but is also expressed on a subset of T cells, where it plays a role in modulating immune responses, often through inhibitory signals (14). These receptors play a crucial role in regulating immune responses by providing inhibitory signals that help to balance immune activity and prevent excessive tissue damage (15, 16). Pioneering research on SARS-CoV-1 infection revealed that both the number of circulating NK cells and the expression of inhibitory KIR CD158b were lower compared to those observed in healthy individuals and patients with Mycoplasma pneumoniae infection. This reduction was linked to greater disease severity and the presence of antibodies specific to SARS-CoV-1 (17). In the context of COVID-19, CD158b and its associated receptors are of interest, as SARS-CoV-2 induces intricate immune responses that engage both innate and adaptive immunity. NK cells, which express KIRs like CD158b, are vital to the innate immune system and are instrumental in the initial response to viral infections (18). CD158b has been found at lower frequency in acute phase patients with severe COVID-19 (18). However, the longitudinal role of CD158b receptors in T cells post SARS-CoV-2 infection has not been investigated.

During infection, antigen presenting cells (APCs) present short peptide sequences known as epitopes to T cell receptors (TCRs) via class I and class II major histocompatibility complex (MHC) proteins. Class I MHC proteins are found on the surface of all nucleated cells and present epitopes of 8-10 amino acids (aa) in length to cytotoxic CD8^+^ T cells. Class II MHC proteins are only present on specialised antigen presenting cells (dendritic cells, macrophages, B cells) and predominantly present to CD4^+^ T cells (19). In both cases the peptide MHC (pMHC) interacts with TCRs in a conserved diagonal docking modality that is believed to have arisen from co-evolution of TCR and MHC genes (20, 21).

TCRs are dimeric structures comprised of either alpha-beta (αβ) or gamma-delta (γδ) chains. αβ chains are the dominant subgroup and are found on ≥95% of all T lymphocytes and are formed through somatic recombination of gene segments during thymocyte development (22, 23). There are a total of 70 variable (V) and 61 joining (J) genes involved in α chain rearrangement and 52 V and 13 J genes alongside 2 diversity (D) genes in β chain rearrangement (24). This process, known as V(D)J recombination can theoretically give rise to more than 2.5 x 10^7^ unique TCRs (25), yet due to MHC-Restriction we often identify overlaps in TCR repertoires for a variety of foreign and self-antigens that can provide valuable insights into previous exposure and persistence of protective immunity.

**Figure 1:**
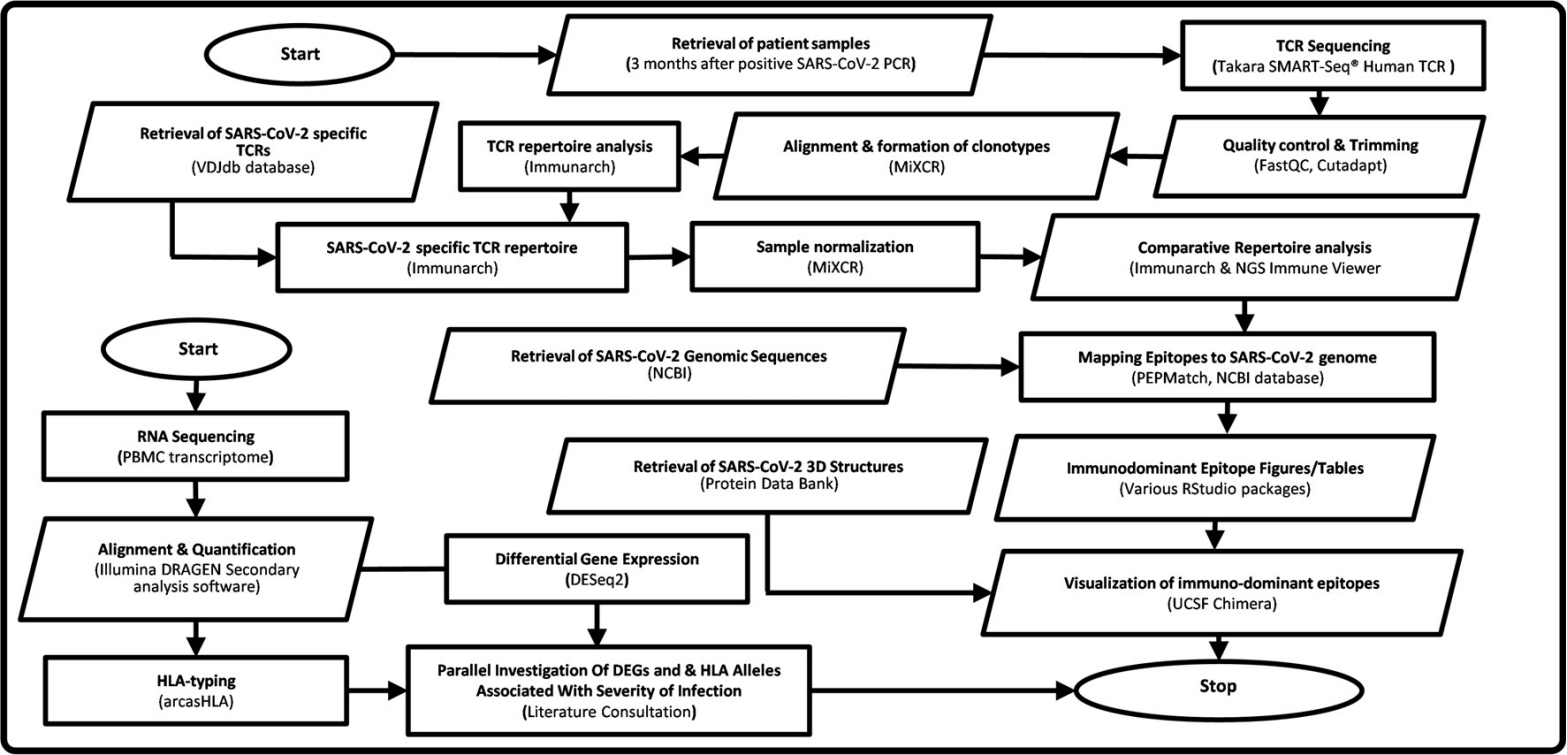
Flowchart of Epi-TAP: Epitope Target Analysis Pipeline (Version 1.0). Oval shapes represent start/stop of the pipeline. Parallelogram boxes represent input/output. Rectangular boxes represent processing steps. Servers/tools/software and databases used are mentioned in parenthesis.

In this paper we investigate persistence of T cell based protective immunity by restimulation with SARS-CoV-2 peptide pools and comprehensive TCR repertoire analysis, in hospitalised and non-hospitalised subgroups. Here we describe a novel immuno-informatics pipeline for TCR repertoire analysis using publicly available tools, for rapid identification and investigation of epitope presentation and clonotype formation. Our novel Epitope Target Analysis Pipeline (Epi-TAP) is capable of discerning unique biological features between subgroups including the presence of immunodominant epitopes, meta-clonotype analysis, severity associated HLA-types, differential expressed genes (DEGs) and pathway activation following viral peptide stimulation. This comprehensive approach could aid in developing future diagnostic and therapeutic strategies against SARS-CoV-2 and future pathogens (**Figure 1**).

## Methods

### Participant inclusion criteria and ethical approval

The following inclusion criteria were used for the current study: 1) Participants were ≥18 years of age at time of recruitment, with any body mass index (BMI) or ethnicity, 2) All participants had a positive PCR test for SARS-CoV-2 at recruitment, 3) Non-hospitalised participants recovered from acute infection without admission to hospital, 4) Hospitalised participants were admitted to hospital during the acute infection, 5) Participants were excluded if <18 years of age or with intellectual disabilities or mental health illness. Ethical approval was obtained from the Health and Care Research Wales Ethics committee (20/WA/0179; 14/07/2020). Informed consent was obtained for all participants in the study, allowing for publication of anonymised demographic and clinical data. The COVID-19 Research Study (COVRES) protocol has further details on study design, data and sample collection and analyses performed (ClinicalTrials.gov NCT05548829) (26).

### Participant recruitment

Participants (n=20) were recruited between October 2021 - May 2022 during which the predominant variant of concern detected in UK during this period was Omicron B.1.1.529 (which includes sub lineages BA.1, BA.2, BA.4 and BA.5).

### Sample collection and processing

Blood was collected in EDTA tubes using 21G Vacuette^®^ safety needles (Greiner Bio-One Ltd, UK) and processed no more than 2 hours post blood draw in a biosafety level 2 laboratory. Peripheral blood mononuclear cells (PBMCs) were isolated using the ficoll gradient separation method. Whole blood collected in EDTA tubes, 18ml was separated using ficoll-plaque plus (Cytiva, UK) by spinning at 400 x g for 30 min, and PBMCs were collected at the interface layer. Cells were washed and counted for recovery and viability using trypan blue, the Countess Automated Cell Counter and slides as per manufacturer instructions (Thermo Scientific, UK). Aliquots of PBMCs were cryopreserved in human serum + 10% DMSO (Sigma-Aldrich; UK) at -80°C for 24 h then transferred to liquid nitrogen for long term storage.

### Full blood counts

A sample of whole blood (WB) was also used to measure full blood counts (FBC)with a standard flow detection Sysmex XE-instrument in the pathology laboratories at Altnagelvin Area Hospital, Londonderry. Clinical data were recovered from the Northern Ireland Electronic Care Records (NIECR).

### Omicron peptide pool and peripheral blood mononuclear cell culture

Peripheral blood mononuclear cells (PBMCs) were cultured and stimulated *in vitro*. Briefly, the cryopreserved PBMCs were thawed and washed, followed by seeding at 1x10^6^ cells per well in RPMI + 5% human AB serum (Gibco, Thermo Scientific, UK). Cells were stimulated with a cocktail of peptides consisting of SARS-CoV Omicron variant Prot_S B.1.1.529/BA.1 Mutation pool (#130-129-928) and SARS-CoV Wuhan wild-type Prot_N (#130-126-698) (Miltenyi Biotec, UK) at a concentration of 1µg/ml of each peptide pool in RPMI + 5% AB serum for 24 hrs, after which cells were harvested and analysed by immunocytochemistry. Cells treated with no peptide (unstimulated cells) were used for baseline negative control readings. Cells treated with phytohemagglutinin (PHA) were included as a positive control. Cell viability was assessed using trypan blue staining method.

### Flow cytometry cell staining and analysis

PBMCs harvested from *in vitro* experiments were stained with fluorophore tagged monoclonal antibodies. Two antibody panels (TBNK and COVRES2) were used for this analysis (Ig isotype, dye, clone and product # are listed for each). TBNK: FITC mouse IgG2a anti-human CD3 (clone OKT3, #566783), PE-Cy7 mouse IgG1 anti-human CD4 (clone SK3 #557852), APC-Cy7 mouse IgG1 anti-human CD8 (clone SK1, #557834), PE mouse IgG1 anti-human CD16 (clone 3G8 #555407), APC mouse IgG1 anti-human CD19 (clone HIB19 #555415), PerCP-Cy5.5 mouse IgG1 anti-human CD45 (clone HI30, #564105), PE mouse IgG1 anti-human CD56 (clone B159 #555516). COVRES2: FITC mouse IgG2a anti-human CD3 (clone OKT3, #566783), APC mouse IgG1 anti-human CD4 (clone SK3, #566915), BV605 mouse IgG1 anti-human CD8 (clone SK1, #564116), R718 mouse IgG1 anti-human CD16 (clone 3G8, #566970), PE-Cy7 mouse IgG1 anti-human CD56 (clone B159, #557747), BV421 mouse IgG2b anti-human CD45RA (clone HI100, #562885), APC-H7 mouse IgG2b anti-human CD45RO (clone UCHL1, #561137), BB700 mouse IgG2b anti-human CD158b (clone CH-L, #561137), PE mouse IgG1 anti-human IL-2 (clone 5344.111, #569370) antibodies, before analysis by a FACS Aria III instrument (all BD Biosciences, UK). The FACS Aria III was operated using FACSDiva software (v9.4; BD). One million (1 x 10^6^) cells in staining buffer were stained with above antibodies according to the manufacturer’s instructions. For surface staining, cells were incubated with relevant fluorochrome-labelled antibodies for 30 min at room temperature in the dark. For cells to be both surface and intracellular stained, prior to staining, these cells were also incubated with a transport inhibitor, BD GolgiPlug™ containing brefeldin A, for 4 hrs at 37°C, BD Cytofix/Cytoperm Plus kit (BD Biosciences, UK), following surface staining cells were fixed and permeabilised using the BD Cytofix/Cytoperm Plus kit (BD Biosciences, UK) and stained with PE-anti-IL-2 for 30 min at 4°C in the dark. All samples were analysed immediately after staining. In each instance, 10,000 events were recorded in triplicate for each individual well/sample. Antibody isotype control, unstained control, cell viability control (see cell culture above) were included in each experiment. Isotype controls were Ig isotype and fluorophore matched antibodies raised against non-human target; viability control was cell samples stained by trypan blue.

### Flow cytometry gating strategy

**Figure 2:**
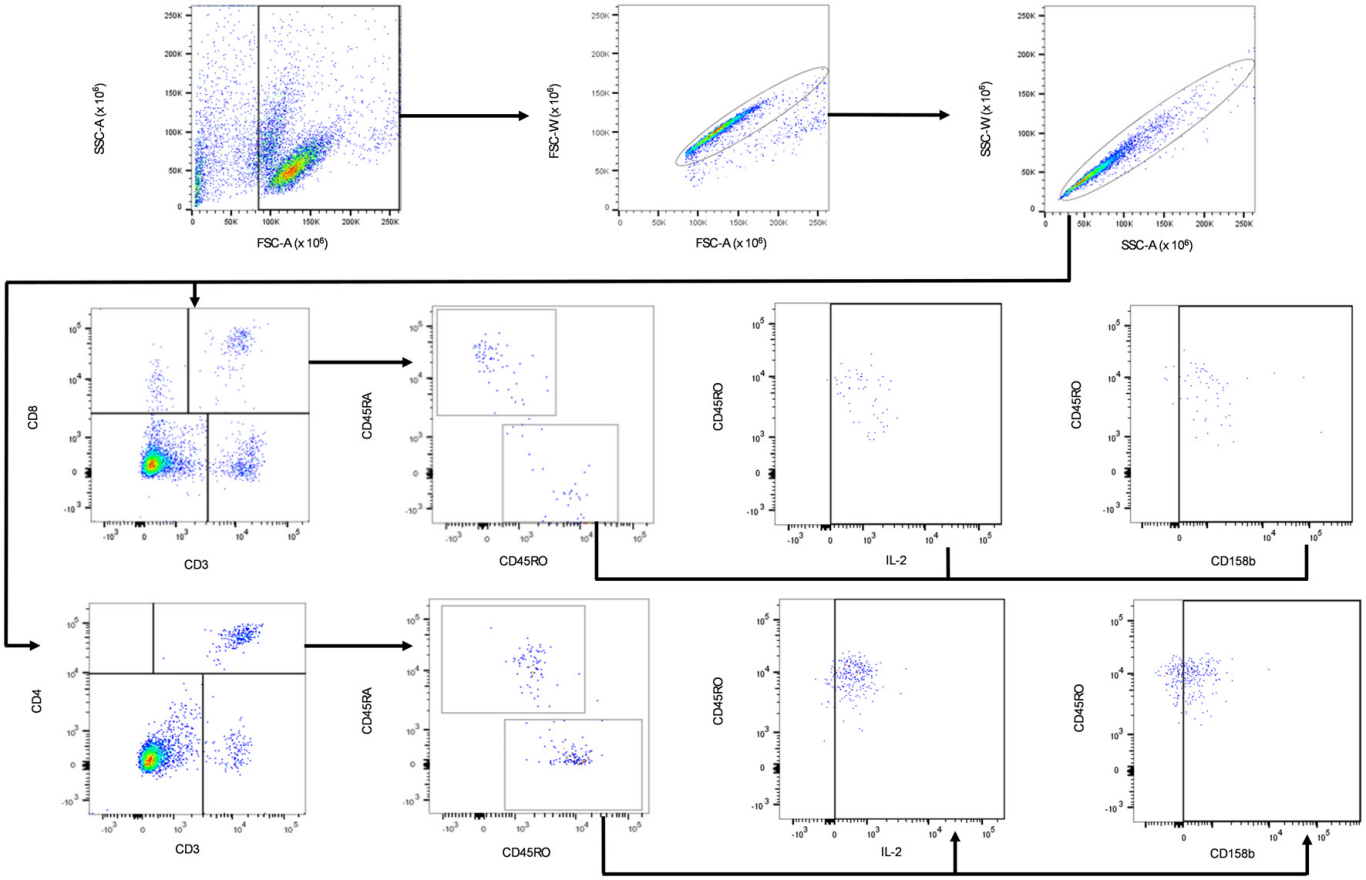
Gating strategy for the identification of IL-2 and CD158b positive CD4^+^CD45RO^+^ and CD8^+^CD45RO^+^ T. Black arrows show the sequence of gates applied to cell subpopulations. Lymphocytes were gated based on the low SSC-A and FSC-A subpopulation within PBMCs. The singlet lymphocytes were gated by a sequence of FSC and SSC sub-gates. CD3^+^ T lymphocyte-specific marker was used to gate for CD3^+^CD4^+^ and CD3^+^CD8^+^ cells. Activated cells were identified by further gating on the CD3^+^CD4^+^CD45RO^+^ and CD3^+^CD8^+^CD45RO^+^ cell subpopulations. CD3^+^CD4^+^CD45RO^+^ and CD3^+^CD8^+^CD45RO^+^ T cells were further sub-gated with IL-2 and CD158b expression.

The gating strategy for flow cytometry analysis is shown in **Figure 2**. Lymphocyte populations were gated based on forward scatter area (FSC-A) and low side scatter area (SSC-A). Where the FCS-A event size was less than 80,000 the population was predominantly cell debris and excluded from analysis. The lymphocyte population was further gated by duplet exclusion (FSC-A, area vs FSC-W, width and SSC-A, area vs SSC-W, width). The T cell subpopulations were identified based on T cells specific markers, T helper (CD3^+^CD4^+^) and cytotoxic T cell (CD3^+^CD8^+^) markers. The T helper and cytotoxic T cell populations were further gated for CD45RO^+^ (memory) T cells. CD45RO^+^ T helper and cytotoxic T cell populations were further gated for CD158b^+^ cell surface and intracellular IL-2^+^ expression, as shown in **Figure 2**.

### Procedure for fluorochrome spillover compensation

To calculate the compensation matrix and to correct the reciprocal spillover among fluorochromes with overlapping emission spectra, anti-mouse (#552843) Ig-Kappa Comp Beads were stained accordingly to the manufacturer’s conditions with the same mouse Igk anti-human antibodies used in the panels and immediately acquired using BD FACSDiva software (BD Biosciences version 9.4). The compensation matrix was automatically calculated by BD FACSDiva software. FACSAria III flow cytometer performance was validated daily with BD FACSDiva CS&T research beads (#655051) following the manufacturer’s instructions (BD Biosciences, UK).

### T cell receptor sequencing

Cryopreserved PBMCs were thawed and washed in Mg2^+^ and Ca2^+^ free PBS prior to RNA extraction (Qiagen RNeasy RNA extraction kit). Bulk unpaired TCR sequencing was performed using an Illumina platform with the TakaraBio SMART-Seq Human TCR (with UMIs) and Unique Dual Index (UDI) kits (Cat. 634779, 634725).Total RNA concentrations were calculated using Qubit™ RNA High Sensitivity (HS) kits (Q10210). Sample integrity was investigated using Agilent Fragment Analyzer - High Sensitivity RNA kit (DNF-472-0500). Total RNA concentrations between 2-40ng were used for library preparation using the Illumina Stranded mRNA Prep Ligation kit. PCR amplification was run for a total of 20 cycles after which libraries were normalised to account for variations in concentration prior to pooling.

### Quality control & adapter trimming

FastQC (v 0.11.9) (27) was first used to check the quality of sequencing output files and identify the threshold required to remove poor quality reads and truncated sequences. Cutadapt (v 4.8) (28) was then used to remove adapters flagged by FastQC, trim sequences based on their length and quality and remove poor quality reads and truncated sequences. Sequences <200bp long were removed as downstream analysis requires full coverage of the V(D)J region for alignment and clonotype assembly. Following trimming FastQC was once again used to ensure all unwanted adapters and poor-quality reads were removed.

### TCR repertoire assembly

Repertoire assembly was performed using MiXCR immune profiling software (v 4.4.2) (29). Paired-end fastq files were analysed using the built-in analyse preset for the SMART-Seq Human TCR (with UMIs) kit. The preset leverages the SMART (Switching mechanism at 5’ end of RNA Template) and unique molecular identifiers (UMIs) to capture complete V(D)J variable regions for α and β chains (αβTCRs) for accurate clonotype assembly and quantification. MiXCR default parameters were used for alignment, PCR error correction and clonotype assembly. For samples with off target (non TCR) reads totalling >10%, alignment was repeated with mix-in options to keep non CDR3 alignments and export non-aligned sequences. Seqtk (v 1.4) (30) was then used to convert output fastq files to fasta format for compatibility with National Center for Biotechnology Information (NCBI) databases (31). Fasta sequences were analysed using the basic local alignment search tool (BLAST) to investigate the origin of non TCR reads. After implementing the above described post-alignment quality control checks, productive clones were exported for αβTCRs in tsv format for downstream analysis.

### SARS-CoV-2 TCR repertoire analysis

A variety of software packages and databases were used to investigate the presence of SARS-CoV-2 specific αβTCRs (CoV-TCRs) and explore shared and unique features between subgroups (**Figure 1**). A number of databases were considered prior to analysis including ImmuneCODE™, Pan immune repertoire database (PIRD), McPAS-TCR and VDJdb (32–35). ImmuneCODE™ was disregarded as it focuses solely on TCRβ repertoires and was not natively compatible with our immunoinformatics pipeline. PIRD and McPAS-TCR datasets were compatible, however PIRD lacked SARS-CoV-2 specific TCRs and McPAS-TCR contained <200 CoV-TCRs at the time of analysis. VDJdb was both compatible with our pipeline and contained a total of 10,080 CoV-TCRs at the time of analysis. First, publicly available CoV-TCRs were retrieved from the VDJdb browser and imported into RStudio (36) using the Immunarch package (37). Output files from MiXCR analysis were also imported using Immunarch and matched against VDJdb using complementary determining region 3 (CDR3) for both α and β chains to identify the presence of CoV-TCRs. Repertoires that reported CoV-TCRs were taken forward for further analysis. Samples were first downsampled in MiXCR to remove redundant data and account for sequencing bias between samples. Automatic downsampling (with no threshold set in MiXCR) was performed using the number of reads from the smallest sample, with productive clones exported for further analysis. Following normalisation samples were again imported into RStudio for comparative analysis. TCR repertoires were mapped against VDJbd separately for α and β chains using a custom function that reduced each individual’s repertoire to include only TCRs that had an exact match with CDR3 sequences retrieved from VDJbd. An initial investigation of clone counts, clonotype abundance, repertoire overlap, CDR3 length distribution and Kmer analysis of CoV-TCRs was then preformed in RStudio using the Immunarch package.

Repertoire tables were updated in RStudio using a custom function that appended the epitope targeted by the CDR3 region to each. A custom function was then used to combine the repertoire results of each subgroup and extract relevant data to investigate the possibility of unique repertoire features, including chain distribution and pairing, meta-clonotype analysis and presence of immunodominant epitopes. Clonotypes were defined as having a unique clonal sequence at the nucleotide level. If a clonal sequence was observed across multiple individuals, sample data was retained and read counts were combined to accurately calculate the number of individuals targeting each epitope, alongside the overall fraction of the repertoire each epitope represented. The number of individuals with CoV-TCRs targeting each epitope, alongside the number of clonotypes generated for that epitope and percentage of the repertoire they represent were used to investigate the presence of immunodominant epitopes (>5% of total repertoire for either subgroup). Immunodominant epitope tables were exported from RStudio in NGS Immune Viewer (38) compatible format to create chord diagrams for V/J gene pairing and distribution charts. We further investigated differences in gene pairing and distribution by performing two sample t-tests for reported V J genes, using the Benjamini & Hochberg method for false discovery rate correction.

The Protein Data Bank (39) was used to retrieve structural files (Nucleocapsid:8FG2, NSP8:7JLT, NSP3:6WUU, pre-fusion spike:7TGW, post-fusion spike:8FDW) to visualise immunodominant epitopes using UCSF Chimera and obtain the position of structural domains for downstream analysis. PEPMatch (40) was used to determine the position of each epitope in the Omicron BA.2 variant genome (NCBI Accession: OR575624.1) (31) to chart the proteins and structural domains targeted by CoV-TCRs. Results tables then were compiled, detailing the number of individuals and clonotypes targeting each epitope along the SARS-CoV-2 genome and imported into RStudio (36) to visualise the data in bar-chart format.

### RNA-Seq analysis

RNA sequencing was performed on PBMC samples using the Illumina Stranded mRNA Prep Ligation kit. Illumina DRAGEN software (V3.10.12) was used for upstream analysis of fastq files. Sequences were first aligned (human GRCh38_alt_aware reference genome) and annotated (Gencode release 44 GRCh38.p14) for comprehensive gene annotation of reference chromosomes, scaffolds, assembly patches and alternate loci (haplotypes).

DESeq2 (41) was used to identify differentially expressed genes (DEGs). Raw counts and metadata were imported to RStudio with the non-hospitalised subgroup used as the reference level. Following normalisation differential expression analysis was carried out on protein coding genes. Genes were classified as differentially expressed if they had a p-adjusted value <0.05. Volcano plots and heatmaps for DEGs were then plotted in RStudio using Pheatmap (42) and EnhancedVolcano (43) packages, incorporating hierarchical clustering of both samples and DEGs. Gene ontology (GO) analysis was performed on DEGs using clusterProfiler (44) with a relaxed p-adjusted value <0.10 for biological processes. Enriched GO terms were then plotted using Enrichplot (45) to visualise the relationship between enriched pathways using pathway enrichment and directed acyclic graphs. Output fastq files from RNA-Seq analysis were also subjected to HLA typing using arcasHLA (46). The frequency of HLA genes was then calculated to assess variance between subgroups and how it might affect antigen presentation and T cell differentiation.

### Data and statistical analysis

Demographic data statistical analyses were performed using IBM SPSS statistics 28.0.1.1. Chi square test was used to compare differences between groups.

GraphPad Prism software (version 9.4.0) was used to analyse full blood count data. T-tests with Welch’s t-tests were used to test differences between groups.

**Figure 3:**
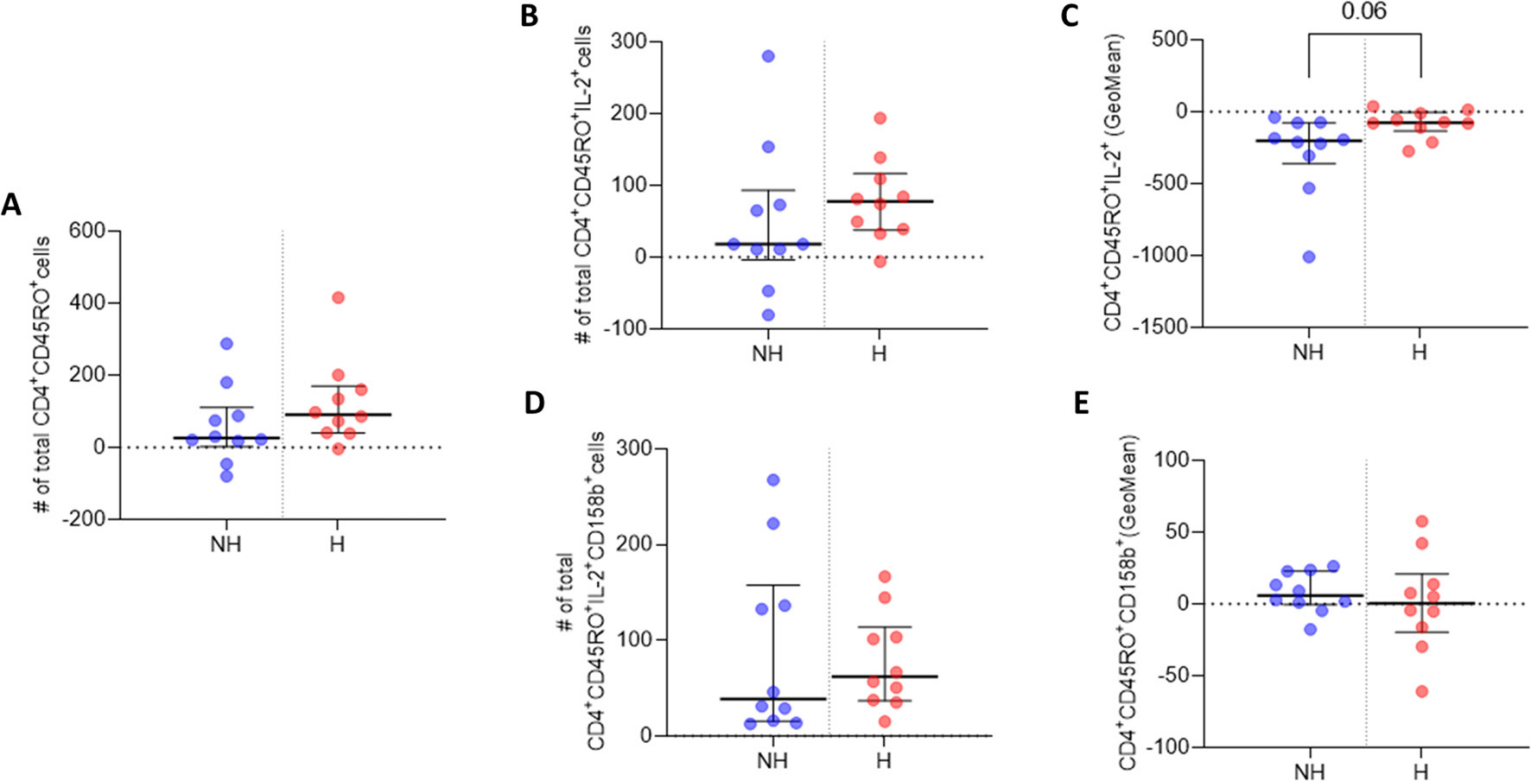
IL-2 and 158b expression in CD4^+^CD45RO^+^ T cells. **(A)** Median number of CD4^+^CD45RO^+^ T cells. **(B)** Median number of CD4^+^CD45RO^+^IL-2^+^ T cell events. **(C)** GeoMean of IL-2 expression in CD4^+^CD45RO^+^IL-2^+^ T cells. **(D)** Median number of CD4^+^CD45RO^+^158b^+^ T cell events. **(E)** GeoMean of 158b expression in CD4^+^CD45RO^+^158b^+^ T cells. All data are presented as Delta values with median and IQR for a given number of observations (n=10 non-hospitalised, n=10 hospitalised). Welch’s t-tests were used for statistical analysis. Data was considered to be significant if p <0.05.

**Figure 4:**
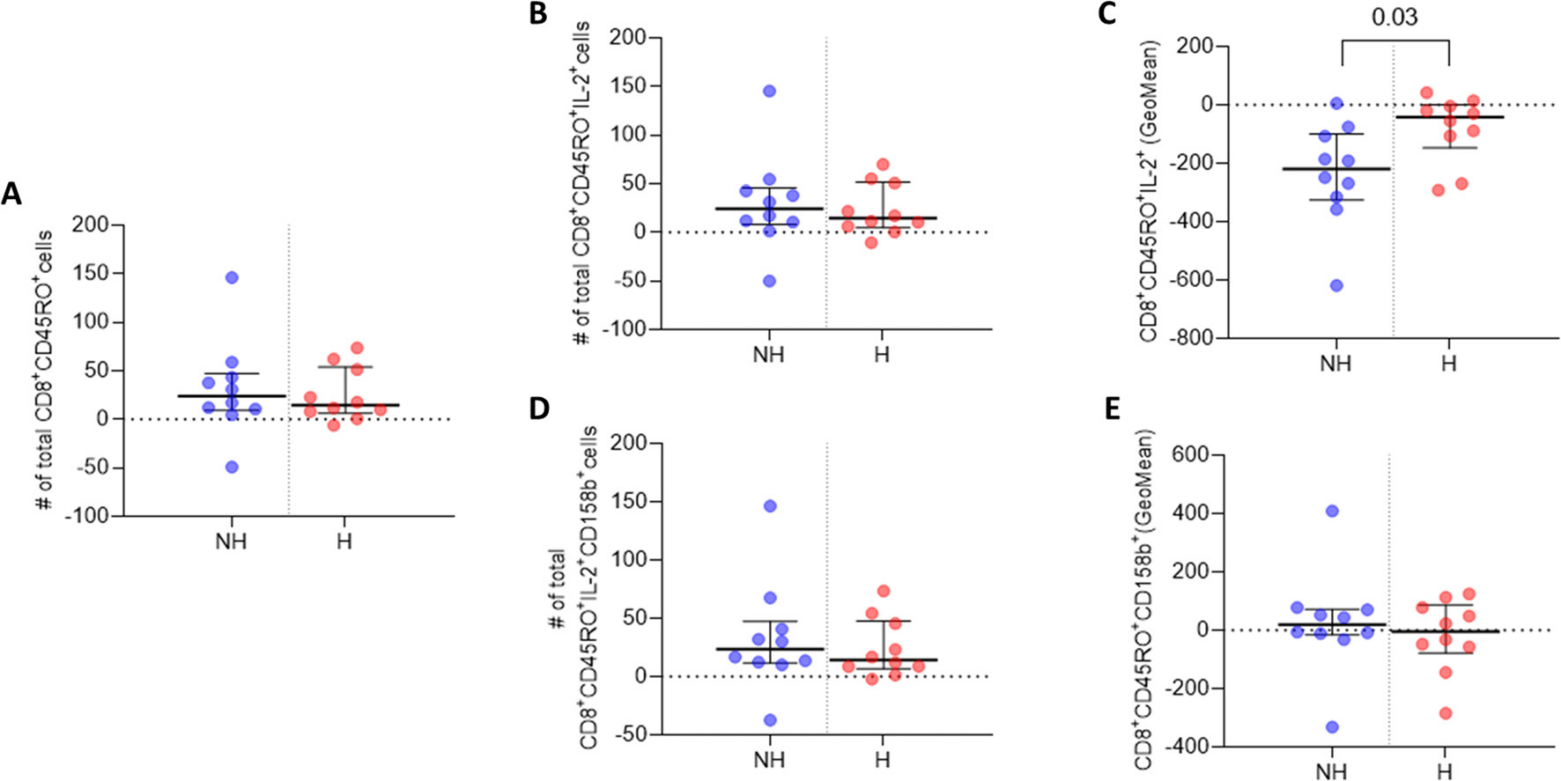
IL-2 and 158b expression in CD8^+^CD45RO^+^ T cells. **(A)** Median number of CD8^+^CD45RO^+^ T cells. **(B)** Median number of CD8^+^CD45RO^+^IL-2^+^ T cell events. **(C)** GeoMean of IL-2 expression in CD8^+^CD45RO^+^IL-2^+^ T cells. **(D)** Median number of CD8^+^CD45RO^+^158b^+^ T cell events. **(E)** GeoMean of 158b expression in CD8^+^CD45RO^+^158b^+^ T cells. All data are presented as Delta values with median and IQR for a given number of observations (n=10 non-hospitalised, n=10 hospitalised). Welch’s t-tests were used for statistical analysis. Data was considered to be significant if p <0.05.

Flow cytometry data analysis and TBNK counts were performed using FlowJo Software (version 10.8.1) (BD Biosciences, UK) on live cells after the exclusion of cell doublets (**Figure 2**). Manual gating for flow cytometry memory T cell data analysis was performed using FlowJo Software (version 10.8.1) (BD Biosciences, UK) on live cells after the exclusion of cell doublets (**Figure 2**), data for each participant was harvested and GraphPad Prism software (version 9.4.0) was used to generate scatter dot plots. For each of n=2 biological replicates per patient, one million PBMCs were isolated from whole blood and antibody labelled, then analysed by flow cytometry with ten thousand events recorded per technical replicate (n=3). The median and IQR event counts were averaged for each patient from the n=3 technical replicates from each of the n=2 biological replicates. Data from stimulated biological replicates were normalised to respective non-stimulated biological replicates, and the normalised values were used to generate the dot plots in **Figures 3**, **4**. Differences between groups were assessed using Welch’s t-tests.

MiXCR immune profiling software (v 4.4.2) was used for TCR repertoire assembly. Downsampling was performed by calculating the 20^th^ quantile across all clonesets (Q20) and normalising the data based on the minimal sample which is above 0.5*Q20.

TCR repertoire analysis was performed using R (v 4.4.1) and RStudio (2024.04.2). SARS-CoV-2 specific repertoire data was exported in csv format for clonotype and meta-clonotype analysis. Chain pairing and CDR3 length distribution analysis was performed using Cogent NGS Immune Viewer available from: https://www.takarabio.com/products/next-generation-sequencing/bioinformatics-tools/cogent-ngs-immune-viewer.

Differential expression analysis of RNA-Seq data was performed using DESeq2 package using R (v 4.4.1) and RStudio (2024.04.2). DESeq2 utilises negative binomial regression for differential expression analysis, Wald test for significance (p value) and Benjamini-Hochberg for multiple testing correction (p-adjusted value). Z-score normalisation and hierarchical clustering was used to produce heatmaps from differentially expressed genes and hypergeometric distribution was used to test for significance in gene ontology analysis. HLA gene frequency and distribution analysis was performed using arcasHLA (v 3.24.0) which utilises read alignment and quantification to infer HLA alleles from RNA-Seq data.

A detailed list of the libraries and the statistical methods employed by their functions along with the code is available in the following public repository: https://github.com/ShuklaLab/Epi-TAP.

## Results

### Demographics

The study cohort consisted of non-hospitalised (n=10) and hospitalised (n=10) participants; 50% were female, the median age was 46.5 years, ranging between 35 to 73 years. Hospitalised participants had a significantly higher median age of 53 years, versus the non-hospitalised median age of 46 years (p <0.001). There was no significant difference in vaccination status between subgroups, with non-hospitalised (n=9) and hospitalised (n=9) participants reporting vaccination prior to infection. No significant difference in comorbidities were reported between non-hospitalised and hospitalised participants (**Table 1**).

**Table 1:** Demographics of study subgroup. Values are median with interquartile range (IQR) or number (n), or percentage (%), where indicated.

Prospective Omicron cohort demographics	Non-hospitalised (n = 10)	Hospitalised (n = 10)	Total (n = 20)	P-value
Female, n (%)	5 (50)	5 (50)	10 (50)	1
Age at diagnosis: Median (IQR)	46 (4.5)	53 (17.5)	46.5 (12.5)	*<0.001
Over 50 years old, n (%)	1 (10)	6 (60)	7 (35)	0.019
Vaccine status, n (%)	9 (90)	9 (90)	18 (90)	1
Comorbidity				
1. Autoimmune, n (%)	1 (10)	2 (20)	3 (15)	0.531
2. Metabolic, n (%)	3 (30)	3 (30)	6 (30)	1
3. Respiratory, n (%)	2 (20)	3 (30)	5 (25)	0.606
4. Cardiovascular, n (%)	1 (10)	4 (40)	5 (25)	0.121
5. Cancer, n (%)	0 (0)	2 (20)	2 (10)	0.136
6. Gastrointestinal, n (%)	1 (10)	1 (10)	2 (10)	1
7. Musculoskeletal, n (%)	1(10)	2 (20)	3 (15)	0.531

P-values were calculated using chi-square (twin paired) test, with the exception of continuous variables* that used a paired Welch’s t-test.

### Full blood counts

Initially, baseline full blood counts revealed a significant decrease in haemoglobin (p <0.02), haematocrit (p <0.02), and red blood cells (RBC) (p <0.01) among the hospitalised subgroup compared to the non-hospitalised subgroup. However, all other baseline blood markers exhibited no significant differences between the two groups (**Table 2**). After three months, no significant difference in blood counts were observed between the two subgroups (**Table 2**).

**Table 2:** Full blood counts at baseline and three months for non-hospitalised and hospitalised: Values are median number of events counted with IQR for each patient.

Cell type (unit)	Baseline					3 months				
	Non-hospitalised		Hospitalised		P-value	Non-hospitalised		Hospitalised		P-value
	Parameter	IQR	Parameter	IQR		Parameter	IQR	Parameter	IQR	
Haemoglobin (g/L)	141.50	13.75	131.00	14.50	*0.02	142.00	13.00	139.00	6.00	0.33
Haematocrit	0.42	0.02	0.39	0.02	*0.02	0.42	0.03	0.41	0.01	0.30
RBC (x10^12^)	4.78	0.55	4.39	0.40	*0.01	4.75	0.68	4.35	0.11	0.13
MCH (pg)	29.90	1.65	30.95	1.40	0.39	30.80	1.50	31.95	1.90	0.19
MCHC (g/L)	33.7	8.25	33.65	9.75	0.39	33.7	6.00	33.6	7.25	0.83
MCV (fl)	88.65	4.70	91.55	3.78	0.39	90.50	5.20	93.70	5.30	0.18
RDW (fl)	12.70	0.88	13.45	1.25	0.06	12.80	0.60	13.65	1.98	0.90
Platelets (x10^9^/L)	278.50	66.50	240.50	136.75	0.65	302.00	49.00	261.00	64.75	0.74
Total WCC (x10^9^/L)	6.47	2.17	6.11	5.14	0.31	7.19	2.04	7.22	2.93	0.36
Neutrophils (x10^9^/L)	3.44	1.79	3.58	2.57	0.70	4.22	1.62	3.77	1.54	0.92
Lymphocytes(x10^9^/L	1.92	0.40	1.94	1.75	0.33	2.05	0.72	1.86	1.45	0.40
Monocytes (x10^9^/L)	0.57	0.28	0.51	0.22	0.90	0.49	0.22	0.52	0.18	0.68
Basophils (x10^9^/L)	0.03	0.03	0.02	0.02	0.20	0.04	0.02	0.04	0.02	0.23
Eosinophils (x10^9^/L)	0.17	0.19	0.12	0.25	0.93	0.15	0.11	0.17	0.19	0.56

Statistical comparisons between groups were done using two tailed Welch’s t-test. Data was considered to be significant *(highlighted in bold) if p <0.05.

### Flow cytometry analysis, TBNK cell counts

Upon examining the total counts of immune cells in both subgroups at three months, it was observed that the median cell counts of all cell subpopulations aside from B cells were higher in the non-hospitalised subgroup in comparison to the hospitalised subgroup. However, none of these differences were statistically significant (**Table 3**).

**Table 3:** TBNK cell counts at three months for non-hospitalised and hospitalised.

3 months					
Cell type	Non-hospitalised		Hospitalised		P-value
	Median cell counts	IQR	Median cell counts	IQR	
T cells	330.7	766.5	251	537.6	0.964
Cyto T cells	196	205.5	128.7	399.8	0.438
Helper T cells	195.7	483.5	155.3	317.2	0.516
B cells	114.8	144.5	148.8	335.3	0.4
NK cells	164.2	134.2	135	121.3	0.523
NK T cells	27.2	81.1	16	80.3	0.848

Statistical comparisons between groups were done using two tailed Welch’s t-test. Data was considered to be significant if p <0.05.

Values are median number of events counted with IQR for each patient.

### Flow cytometry analysis, memory T cells

CD4^+^ T cell analysis revealed a higher median number of CD4^+^CD45RO^+^ memory T cells three months post-infection in the hospitalised subgroup, compared to non-hospitalised individuals, and a higher median number of CD4^+^CD45RO^+^IL-2^+^ memory T cells was also observed upon Omicron peptide pool stimulation *in vitro* in the hospitalised subgroup (**Figures 3A, B**). The median subgroup values of IL-2 geometric mean fluorescence intensity (GeoMean) in these CD4^+^ memory T cells trended towards a statistically significant increase in per cell IL-2 expression in the hospitalised subgroup (p <0.06; **Figure 3C**).

The hospitalised patient subgroup had a higher median number of CD4^+^CD45RO^+^CD158b^+^IL-2^+^ memory T cells, compared to non-hospitalised individuals (**Figure 3D**). However, the hospitalised subgroup median GeoMean value for CD158b expression per cell was marginally lower than that observed in non-hospitalised individuals (**Figure 3E**).

CD8^+^ T cell analysis indicated that the non-hospitalised subgroup had a higher median number of CD8^+^CD45RO^+^ memory T cells three months post infection, relative to the hospitalised subgroup (**Figure 4A**). A higher median number of CD8^+^CD45RO^+^IL-2^+^ memory T cells was observed in the non-hospitalised subgroup upon Omicron peptide pool stimulation *in vitro* (**Figure 4B**). The median subgroup GeoMean values for IL-2 per cell expression in these CD8^+^ memory T cells was significantly increased in the hospitalised subgroup (p <0.03; **Figure 4C**). The non-hospitalised individuals had a marginally higher median number of CD8_+_CD45RO_+_CD158b_+_ memory T cells, compared to the non-hospitalised subgroup (**Figure 4D**). The non-hospitalised subgroup median GeoMean value for CD158b expression per cell was marginally higher than that observed in hospitalised individuals (**Figure 4E**).

### TCR repertoire assembly

Following repertoire assembly alignment and chain usage, alignment rates were investigated to assess sample quality. Two hospitalised subgroup individuals (H7, H10) had alignment rates <90% and were subjected to further investigation. BLAST alignment uncovered that in most instances sequences aligned to TCR genes but were discarded by MiXCR due to partial coverage and not the product of off-target events or foreign contamination. These sequences were subsequently disregarded with only productive clones exported for further analysis. Following alignment, chain usage rates ranged between 15-30% for TCRα and 70-85% for TCRβ (**Supplementary Figure 1**). Prior to CoV-TCR repertoire analysis we investigated the total clonal abundance for αβTCRs between subgroups. The non-hospitalised subgroup had a 2.41-fold increase in TCRα clones and 2.28-fold increase in TCRβ (**Supplementary Figure 2**).

### Characteristics of CoV-TCRs

**Figure 5:**
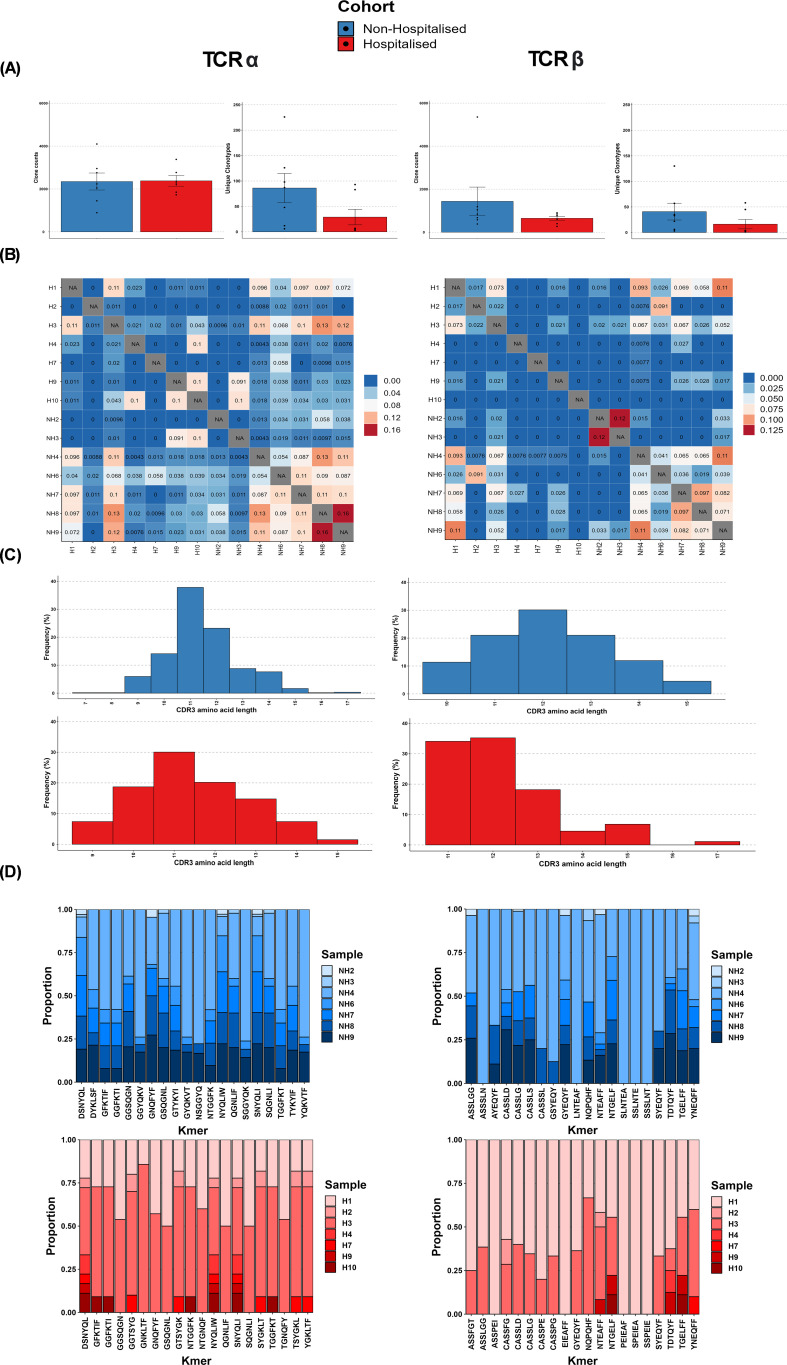
T cell receptor (TCR) repertoire overview. **(A)** Average abundance of SARS-CoV-2 specific clones following immune repertoire analysis. **(B)** Average diversity of SARS-CoV-2 Clonotypes (defined by unique V/J genes CDR3 sequence) **(C)** TCR repertoire overlap (Jaccard similarity index) **(D)** Distribution of CDR3 amino acid length.

Comparative analysis of CoV-TCRs indicated a disparity in both the abundance and diversity of clonotypes between subgroups (**Figure 5A**). Repertoire overlap (**Figure 5B**), calculated using Jaccard similarity index, also showed a higher degree of overlap in the non-hospitalised subgroup (bottom right quadrant) compared to the hospitalised (top left quadrant) for both αβTCR repertoires indicating the presence of meta-clonotypes with shared V genes and CDR3 segments. CDR3 length distribution (**Figure 5C**) for TCRα repertoires is similarly distributed in both subgroups, with peaks at 11 aa in length. The non-hospitalised subgroup sequences ranged between 7-17 aa in length with 75.12% of sequences ranging between 10-12 aa, whereas the hospitalised length ranged between 9-15 with 68.81% of sequences between 10-12 aa in length. CDR3 length ranged between 10-15 aa in the non-hospitalised and 11-17 aa in the hospitalised for TCRβ repertoires. CDR3 length was normally distributed in the non-hospitalised subgroup with 72.03% of sequences in the 11-13 aa range, while the hospitalised subgroup showed a bias for shorter sequence length with 90.35% of sequence between 11-13 aa in length. Kmer analysis (**Figure 5D**) of the top 20 6mers for CDR3 sequence found that the 55% of Kmers overlapped in αβTCR repertoires. This analysis found a disproportionate amount of Kmers for the hospitalised subgroup were reported by two hospitalised subgroup individuals (H1, H3), whereas the non-hospitalised subgroup had a more even distribution of Kmers reported for αβTCR repertoires.

### CoV-TCRs epitope targeting and repertoire diversity

**Figure 6:**
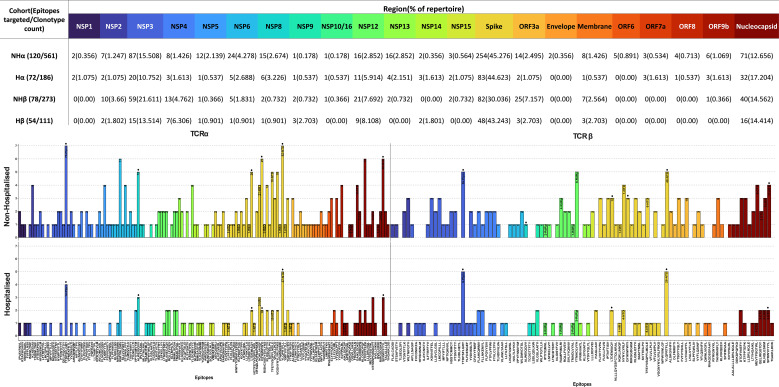
SARS-CoV-2 epitopes targeted by T cell receptor (TCR) repertoire. SARS-CoV-2 epitopes targeted by TCRα and TCRβ chains for Non-Hospitalised (Top) and Hospitalised (Bottom) subgroups. Epitopes are ordered by position along the genome (left to right) and colour-coded by region. The number of clonotypes targeting each epitope are labelled within each bar, alongside their residing structural domain (where applicable). Immunodominant epitopes (>5% of total repertoire from either subgroup) are labelled with a dot above their respective bar.

An investigation into the epitopes targeted by CoV-TCRs (**Figure 6**) found a noticeable increase in both number of epitopes targeted and circulating clonotypes for the non-hospitalised subgroup, with a 1.56-fold increase in targeted epitopes and 3.01-fold increase in clonotypes for the TCRα repertoire and a respective 1.44 and 2.46-fold increase in TCRβ repertoire. An increase in the number of individuals targeting epitopes was observed in the non-hospitalised subgroup. On average 1.94 individuals generated at least 1 clonotype per epitope across αβTCR repertoires compared to an average of 1.39 in the hospitalised subgroup. Overall, the distribution of clonotypes across the SARS-CoV-2 genome was relatively similar between both subgroups, with the majority of clonotypes targeting epitopes in the spike, nucleocapsid and NSP3 regions. The largest disparity in clonotype distribution was found in the spike and NSP3 regions of TCRβ repertoires. The non-hospitalised subgroup had a 1.60-fold increase in distribution of clonotypes for NSP3 whereas the hospitalised subgroup had a 1.44-fold increased distribution of clonotypes for the spike region. Interestingly, despite both subgroups having a heavy bias towards TCRβ chain usage (**Supplementary Figures 1**, **2**) when investigating their complete repertoire, TCRα chain usage was more prevalent in CoV-TCRs.

**Figure 7:**
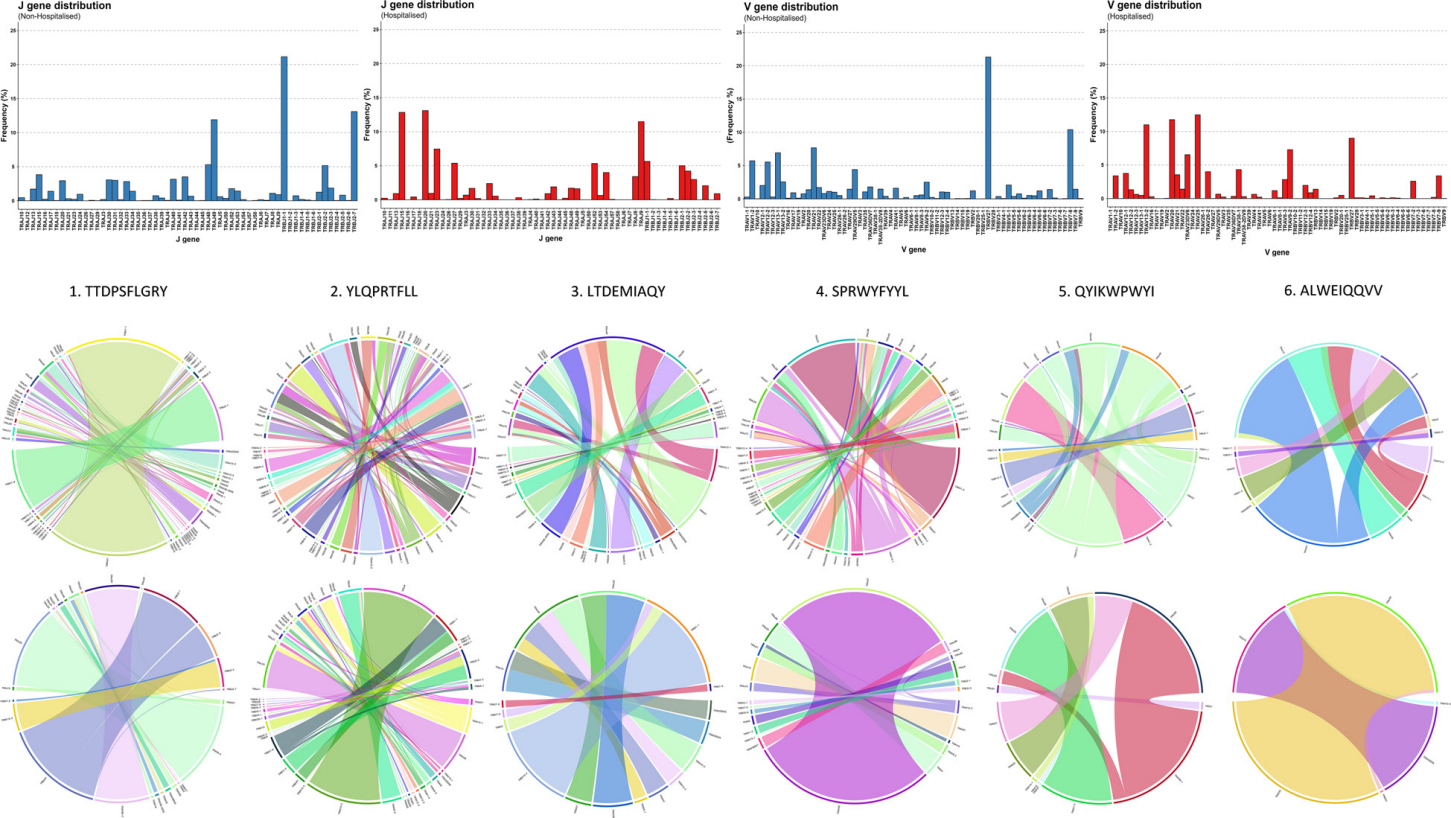
Immunodominant epitope V-J distribution and pairing. Chain distribution and pairing for immunodominant epitopes in Non-Hospitalised and Hospitalised subgroups.

Several immunodominant epitopes (**Supplementary Figure 3**), conserved between both subgroups, were responsible for a large portion of repertoire diversity. Although the immunodominant epitopes were conserved between subgroups, a stark contrast in circulating clonotypes and stereotypy was noted in the non-hospitalised subgroup, with individuals whose HLA-genotype matched the putative HLA alleles for immunodominant epitopes producing a larger variety of clonotypes (**Table 4**, **Supplementary Table 1**). In this context stereotypy is defined as unrelated clones sharing (quasi) identical VDJ rearrangements and CDR3 motifs. V/J gene pairing and distribution (**Figure 7**) further demonstrated that a greater number of individuals producing higher number of clonotypes resulted in convergent recombination of V and J gene segments with a bias towards particular gene pairings for immunodominant epitopes including TRBV27/TRBJ1-1 and TRBV7-8/TRBJ2-7 for epitope 1 (TTDPSFLGRY). Distribution of V/J genes was also markedly different in both subgroups, with the non-hospitalised subgroup having higher proportions of TRAJ1-1, TRAJ2-7 TRAJ49, TRBV7-8 and TRBV27 while the hospitalised subgroup reported higher prevalence of TRAJ9, TRAJ15, TRAJ20, TRAV13-2, TRAV20 and TRAV25. Despite this disparity we found no significant difference in these genes following p-value adjustment of two-sided student’s t-test results.

**Table 4:** Immunodominant epitope results.

Epitope	Cohort										
	Non-hospitalised				Hospitalised				Epitope Information		
	Unique clonotypes	Individuals targeting	% of Repertoire	Dominant pairing	Unique clonotypes	Individuals targeting	% of Repertoire	Dominant pairing	Putative HLA-Alleles	Protein (region)	Conservation (VOI)
(1) TTDPSFLGRY	76	7/7	26.11	TRBV27/TRBJ1-1	20	5/7	11.13	TRAV9-2/TRAJ23	HLA-A*01:01	NSP3	91.79% (123/134)
(2) YLQPRTFLL	100	7/7	13.78	TRAV13-2/TRAJ42	57	5/7	20.06	TRAV13-2/TRAJ9	HLA-A*02:01	Spike(NTD)	100.00% (15/15)
(3) LTDEMIAQY	43	5/7	7.04	TRAV21/TRAJ49	12	2/7	1.53	TRBV12-4/TRBJ1-1	HLA-A*01:01	Spike	100.00% (15/15)
(4) SPRWYFYYL	51	4/7	5.75	TRAV1-2/TRAJ31	15	3/7	4.41	TRAV20/TRAJ27	HLA-B*07:02	Nucleocapsid(NTD)	100.00% (125/125)
(5) QYIKWPWYI	25	6/7	3.31	TRAV1-2/TRAJ33	10	2/7	5.01	TRAV38-1/TRAJ52	HLA-A*24:02	Spike	100.00% (15/15)
(6) ALWEIQQVV	12	5/7	2.22	TRAV21/TRAJ15	4	3/7	9.55	TRAV25/TRAJ20	HLA-A*02:01	NSP8	100.00% (134/134)

Epitopes >5% of the total repertoire from either cohort were considered immunodominant.

Unique clonotypes and dominant pairing were calculated in RStudio using the immunarch package. Putative HLA alleles were retrieved from the VDJdb database. Conservation analysis was performed on sequences retrieved from NCBI database.

### RNA-Seq analysis

RNA-Seq analysis reported a total of 26 protein coding DEGs predominantly activating pathways involved in wound healing, immune cell development and differentiation (p <0.05; **Supplementary Figures 4A, C**). Hierarchical clustering of individual samples uncovered that while the non-hospitalised subgroup clustered in close proximity the hospitalised subgroup had groups at either end of the clustering dendrogram with vastly different expression of certain DEGs (**Supplementary Figure 4B**). Interestingly samples H1 and H3 which clustered with the non-hospitalised subgroup also shared the highest degree of repertoire overlap and Kmer similarity with our non-hospitalised subgroup following TCR repertoire analysis. HLA typing analysis (**Supplementary Table 1**) found that each member of the non-hospitalised subgroup expressed at least one of the putative HLA alleles associated with immunodominant epitopes (**Table 4**) which was only the case for 5/7 participants from the hospitalised subgroup. The overall frequency of expression (**Supplementary Figure 4D**) for MHC class I alleles, including HLA-A and HLA-B genes on which all immunodominant epitopes are presented was found to be higher in the non-hospitalised subgroup. In contrast the hospitalised subgroup reported higher frequencies of MHC class II alleles including HLA-DR alleles.

## Discussion

A comprehensive analysis of CD4^+^ and CD8^+^ memory T cell responses to Omicron spike and nucleocapsid antigens three months after SARS-CoV-2 infection, revealed distinct patterns in protective immune responses across hospitalisation subgroups. In hospitalised COVID-19 patients, an increased frequency of IL-2 producing CD4^+^CD45RO^+^ memory T cells was observed, indicating a persistent CD4^+^ T cell-mediated immune response. Furthermore, within these Omicron specific memory T cells, IL-2 per cell expression was stronger in hospitalised patients. In contrast, CD8^+^CD45RO^+^ T cells producing IL-2 were more frequent in non-hospitalised individuals after re-exposure to Omicron peptide pools. However, per cell IL-2 expression was significantly higher in hospitalised patients. Although hospitalised patients had fewer IL-2 producing memory CD8^+^ T cells, those present produced considerably more IL-2 per cell.

This analysis shows key differences in immune memory responses between subgroups, T cells previously infected and subsequently re-exposed to Omicron peptide pools. Hospitalised individuals exhibited a robust CD4^+^ T cell-mediated response, marked by increased IL-2 production, crucial for memory T cell persistence and immune resilience. These nuanced findings underscore the complexity of memory T cell immunity in response to Omicron acute phase severity and hospitalisation. Weiskopf et al. (9), observed a similar trend, reporting significantly elevated IL-2 levels in the supernatant of *in vitro* peptide-stimulated PBMCs isolated from hospitalised patients 14 days post-admission, though they did not specify which cell subsets were responsible for IL-2 production. Cohen et al. (12), employed comparable methods to investigate CD4^+^ and CD8^+^ T cell memory, using SARS-CoV-2 peptide pools to stimulate PBMCs from COVID-19 patients. Their findings, based on intracellular cytokine analysis, including IL-2, revealed that both T cell subsets retained long-term memory to the virus post-infection. In an acute phase comparison of moderate hospitalised and severe hospitalised cases, Sandberg et al. (47), observed lower absolute numbers of both CD4^+^ and CD8^+^ memory T cells in severe cases. SARS-CoV-2 specific memory T cell frequency to a particular variant appears to peak at 3 months in mild (non-hospitalised) cases (48). Contrary to our observations, at the later time point of 4 months post symptom onset Dan et al. (49), observed that CD4^+^ memory T cell frequencies trended lower in hospitalised cases and CD8^+^ memory T cell frequencies did not differ significantly between hospitalisation subgroups. It is possible that post-acute phase sample timing, sample size and alternate methodologies between studies have contributed to the differences observed. Similar observations were made in the acute phase frequency of CD4^+^ central and effector memory T cells, which were significantly lower in severe (hospitalised) versus mild (non-hospitalised) COVID-19 cases, whereas CD8^+^ compartment frequencies didn’t differ significantly between subgroups (50).

The analysis of CD158b expression revealed no significant differences in the numbers of CD158b-positive CD4^+^CD45RO^+^ and CD8^+^CD45RO^+^ memory T cells, as well as in single-cell levels of CD158b expression, between the hospitalisation subgroups. However, there was a modest increase in the frequency of CD4^+^CD45RO^+^CD158b^+^ cells in the hospitalised group and a slight increase in CD8^+^CD45RO^+^CD158b^+^ cells in the non-hospitalised group. These findings aligned with the broader patterns observed in the CD4^+^CD45RO^+^ and CD8^+^CD45RO^+^ cell populations.

TCR repertoire analysis found that 7 out of 10 participants from both subgroups retained memory of known CoV-TCRs retrieved from VDJdb (35). The non-hospitalised subgroup had notably higher numbers of circulating clones, with a broader variety of clonotypes and a higher degree of convergent recombination, as shown by our repertoire overlap and Kmer analysis. These findings mirror that which have been reported in similar investigations that have associated improved clinical outcome and persistence of protective immunity with increased clonal diversity towards specific epitopes with particular variable and CDR3 segments (51, 52). We also reported a bias towards shorter CDR3 lengths in the hospitalised subgroup in our TCRβ analysis. Germline V(D)J recombination of particular TRBV and TRBJ segments can result in shortening of the CDR3 loop and is in part influenced by MHC haplotype impacting specificity of pMHC presentation to thymocytes (53, 54). Although we found no statistical difference in TRBV and TRBJ genes we observed a higher prevalence of TRBV27 in the non-hospitalised subgroup which has been associated with longer CDR3 segments targeting immunodominant epitopes in recent studies (55). We found the hospitalised bias towards shorter CDR3 lengths was largely driven by an epitope-specific response to immunodominant epitope YLQPRTFLL, which accounted for over half of the clonotypes generated for the hospitalised TCRβ repertoire. Several COVID-19 studies indicate pMHC presentation of particular epitopes can lead to restricted V gene usage impacting CDR3 motif length (56, 57). Additionally Sureshchandra et al. (58), reported increased CDR3 lengths following both vaccination and natural SARS-CoV-2 infection, with preferentially enriched V genes: TRAV29/DV5, TRBV5-1, TRBV6-5, TRBV11-2, TRBV7-9, TRAV12-2 and TRBV6-2. Our analysis found all but TRBV7-9 were enriched in the non-hospitalised subgroup (**Figure 7**), further highlighting how CDR3 length and chain usage characteristics can be clinically relevant and serve as a potential biomarker for at risk populations.

In our findings we reported that a small number of CD8^+^ immunodominant epitopes conserved between both subgroups were responsible for the vast majority of generated clonotypes. Individuals in the non-hospitalised subgroup generated higher numbers of unique and convergent clonotypes, with a fundamentally different distributions of epitope specific V/J gene pairings. Three of the six immunodominant epitopes resided on spike glycoprotein, one on the nucleocapsid protein, and two on NSP3 and NSP8. Two of these epitopes TTDPSFLGRY (NSP3) and YLQPRTFLL (Spike-NTD) were targeted by all members of the non-hospitalised subgroup, accounting for 39.89% of the entire CoV-TCR repertoire. The same epitopes were heavily targeted by the hospitalised subgroup, accounting for 31.19% of their CoV-TCR repertoire, however only five members of the hospitalised subgroup generated clonotypes with decreased clonal diversity. HLA-typing analysis found more individuals in the non-hospitalised subgroup expressed putative HLA alleles for immunodominant epitopes and also reported higher expression of MHC class I alleles overall. In contrast, two of the hospitalised subgroup lacked HLA alleles associated with immunodominant epitopes and had higher expression of MHC class II HLA-DR alleles which have been associated with poorer clinical outcomes and long covid in recent studies (59, 60). Recently Du et al. (61), found persistently high HLA-DR^+^CD38^+^ CD8^+^ T cell counts were associated with immune dysregulation, systemic inflammation and impaired killing potential in severe COVID-19. Similarly, Santopaolo et al. (62), observed significantly higher HLA-DR and CD38 expression, alongside elevated levels of numerous pro-inflammatory cytokines (IL-4, IL-7, IL-17, TNF-α) in CD4^+^ and CD8^+^ T cells in severe COVID-19 compared to mild and moderate patients, 3 months post infection. These results align with findings from our flow cytometry analysis of increased IL-2 expression in both CD4^+^CD45RO^+^ and CD8^+^CD45RO^+^ memory T cells, alongside RNA-seq analysis which indicated increased HLA-DR allele expression of following in the hospitalised subgroup.

It is important to acknowledge the limitations of the study, including the modest cohort sample size, timing of sample collection, the demographics of the study participants, comorbidities and reinfection history (which was not possible to reliably discern without PCR test data). Additional factors for example could contribute to T cell numbers between hospitalisation subgroups such as age, comorbidities and vaccines or treatments received. TCR repertoire analysis is limited by both the number of COVID-19 sequences available from the VDJdb database at the time of the study and strict inclusion criteria using exact CDR3 matches. Widening the scope of the study to include other databases, such as ImmuneCODE™ and exploring CDR3 sequences with small changes in aa sequence using tools such as TCRmatch (63) could further elucidate links between TCR repertoire and hospitalisation, and potentially identify new SARS-CoV-2 clonotypes and HLA relationships.

At 3 months post COVID-19, we have observed strong and persistent Omicron specific CD4^+^ memory T cells in hospitalised individuals in particular, and a significant proportion of CoV-TCRs targeting epitopes along the SARS-CoV-2 genome. The accentuated per cell IL-2 expression in both CD4+ and C8+ memory T cells of hospitalised patients and altered TCR diversity may indicate the regulation and integrity of T cell based immune protection is compromised over the longer term in individuals previously hospitalised by COVID-19, though this requires confirmation by further longitudinal sampling and analyses. A study of the same cohort revealed that hospitalised patients more frequently experience persistent symptoms in the 3-12 months after Omicron infection (64). The most prevalent persisting symptoms in hospitalised patients were muscle pain and shortness of breath, and they display increased mental health difficulties over time. Furthermore, we observed that hospitalised patients have lower frequency of virus neutralising antibodies and higher proinflammatory protein levels 3 months after infection. This study therefore highlights the importance of considering how thymocyte development and antiviral immune responses are impacted by both HLA-type of patients and the viral lifecycle of SARS-COV-2. The identification of shared immunodominant T cell epitopes in hospitalised and non-hospitalised subgroups that reside outside of the spike protein further highlights the importance of targeting replication proteins in future vaccine strategies.

## Data Availability

The data generated in this study has been deposited in the European Genome-phenome Archive (EGA) and are made findable through the EGA web portal (https://ega-archive.org, study number: EGAD50000001356). Access to the data will be granted upon approval by the Data Access Committee (DAC) and in accordance with the conditions outlined in the Data Access Agreement (DAA).
