# Supplementary material for: Distinct Omicron longitudinal memory T cell profile and T cell receptor repertoire associated with COVID-19 hospitalisation
Source: Front Immunol. 2025 Apr 2;16:1549570. doi: 10.3389/fimmu.2025.1549570 (PMC12000046; doi:10.3389/fimmu.2025.1549570)
Supplement: Supplementary file 1 [file DataSheet1.docx]

Supplementary Material


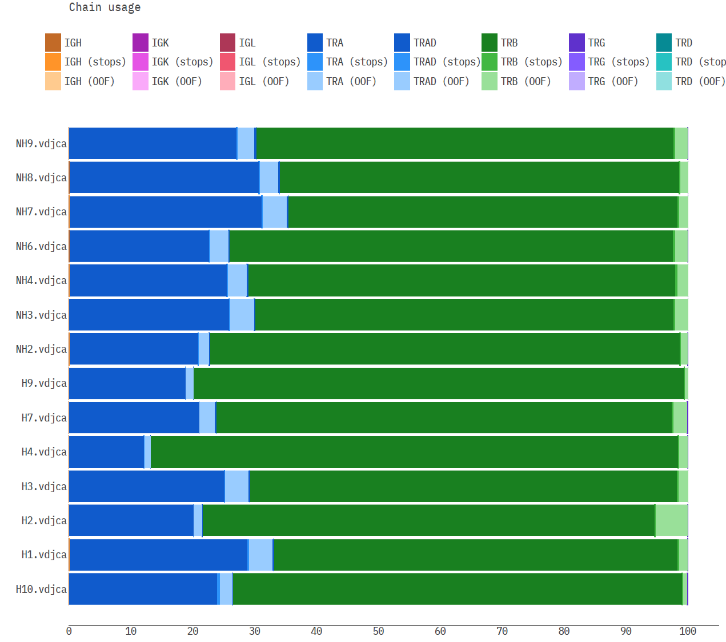

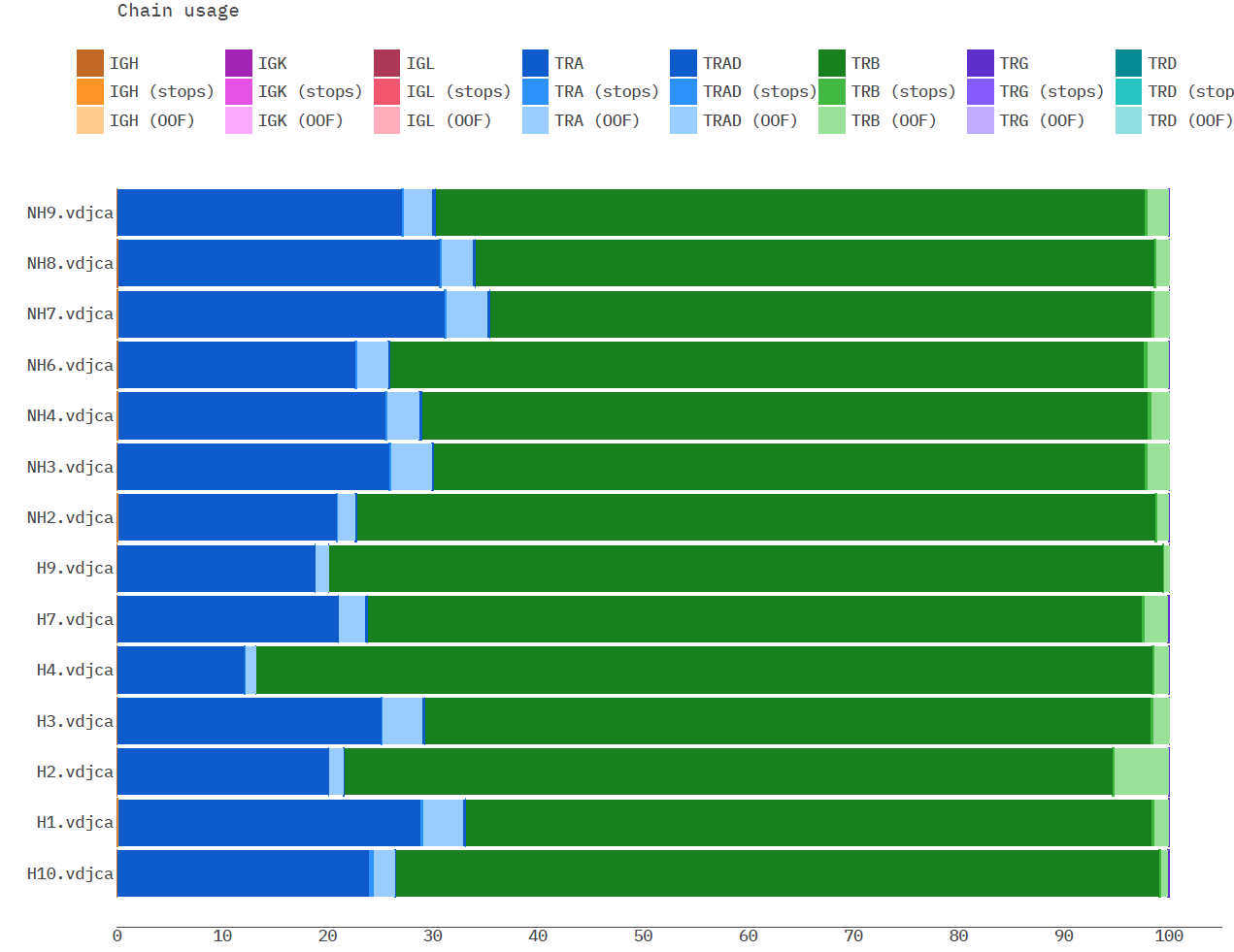

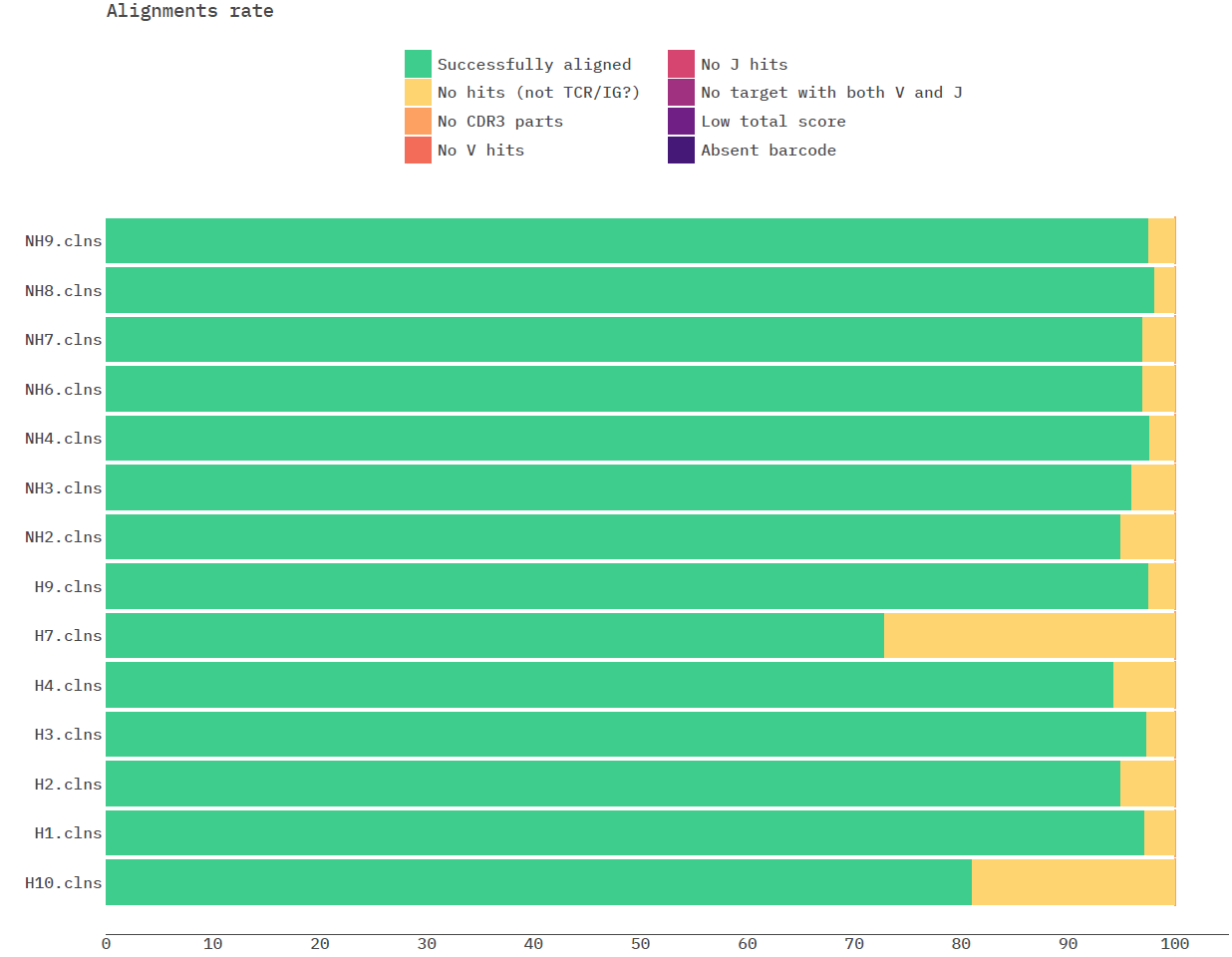

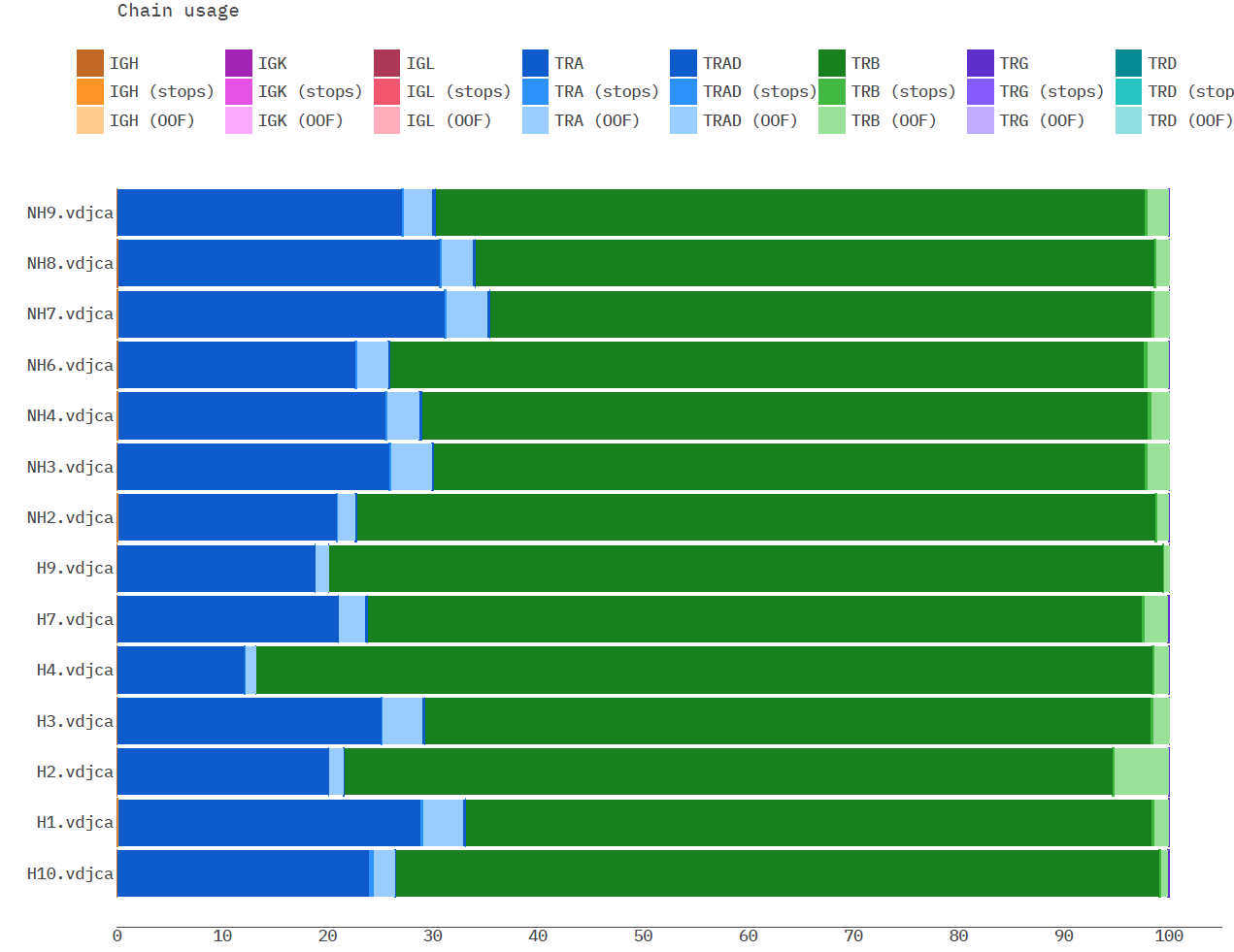

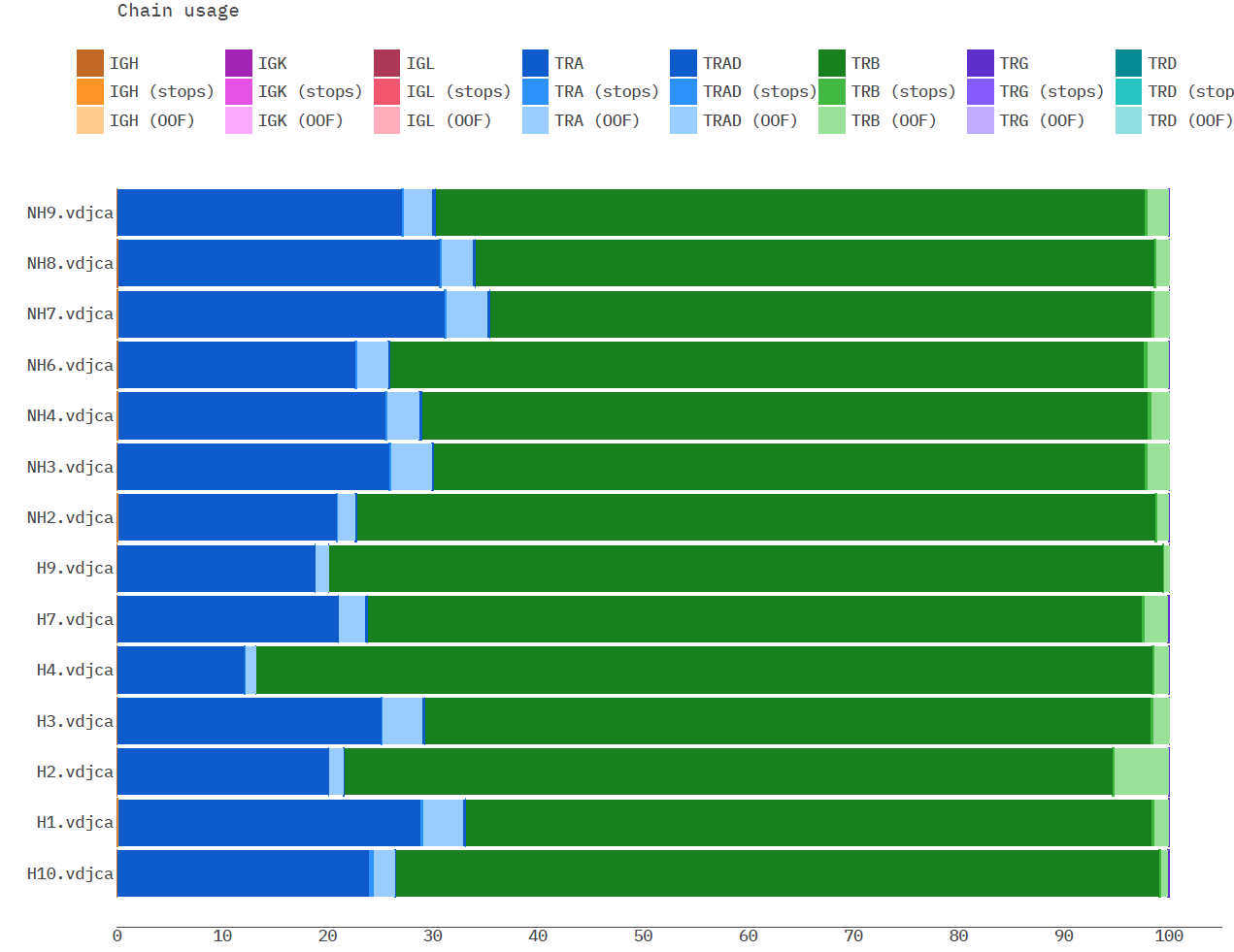


**Supplementary Figure 1: T Cell Receptor (TCR) alignment and chain usage.**Alignment rate and chain usage distribution. Calculated following downsampling in MiXCR for CoV-TCR^+^ samples.


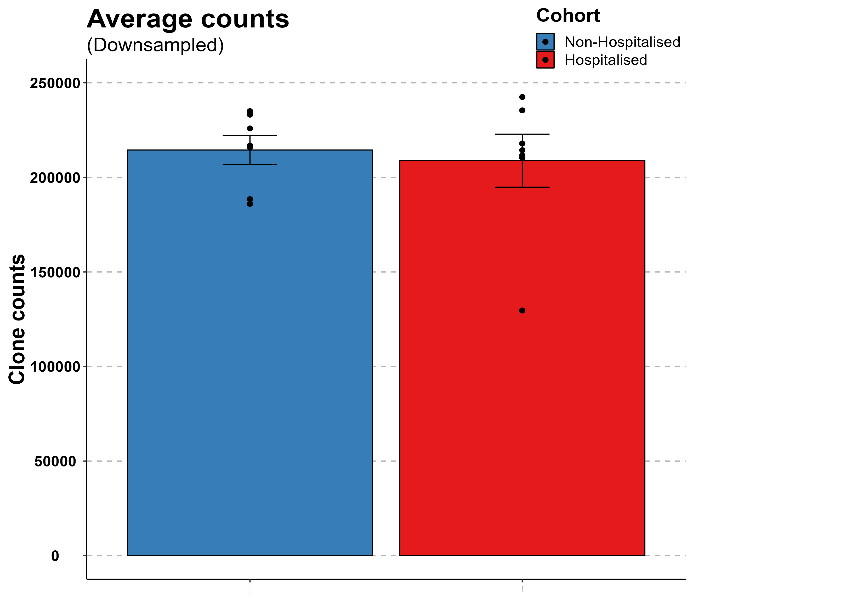

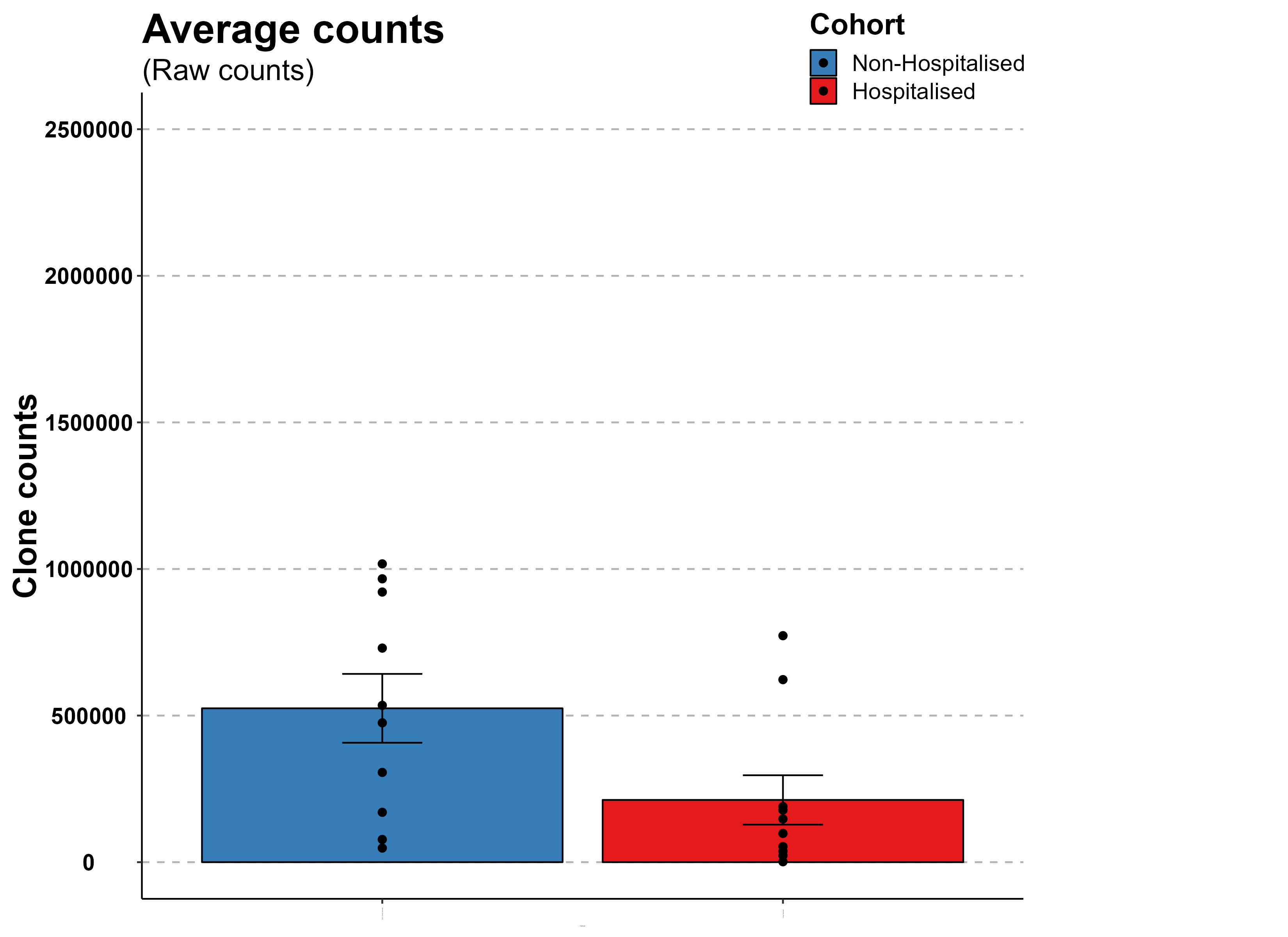

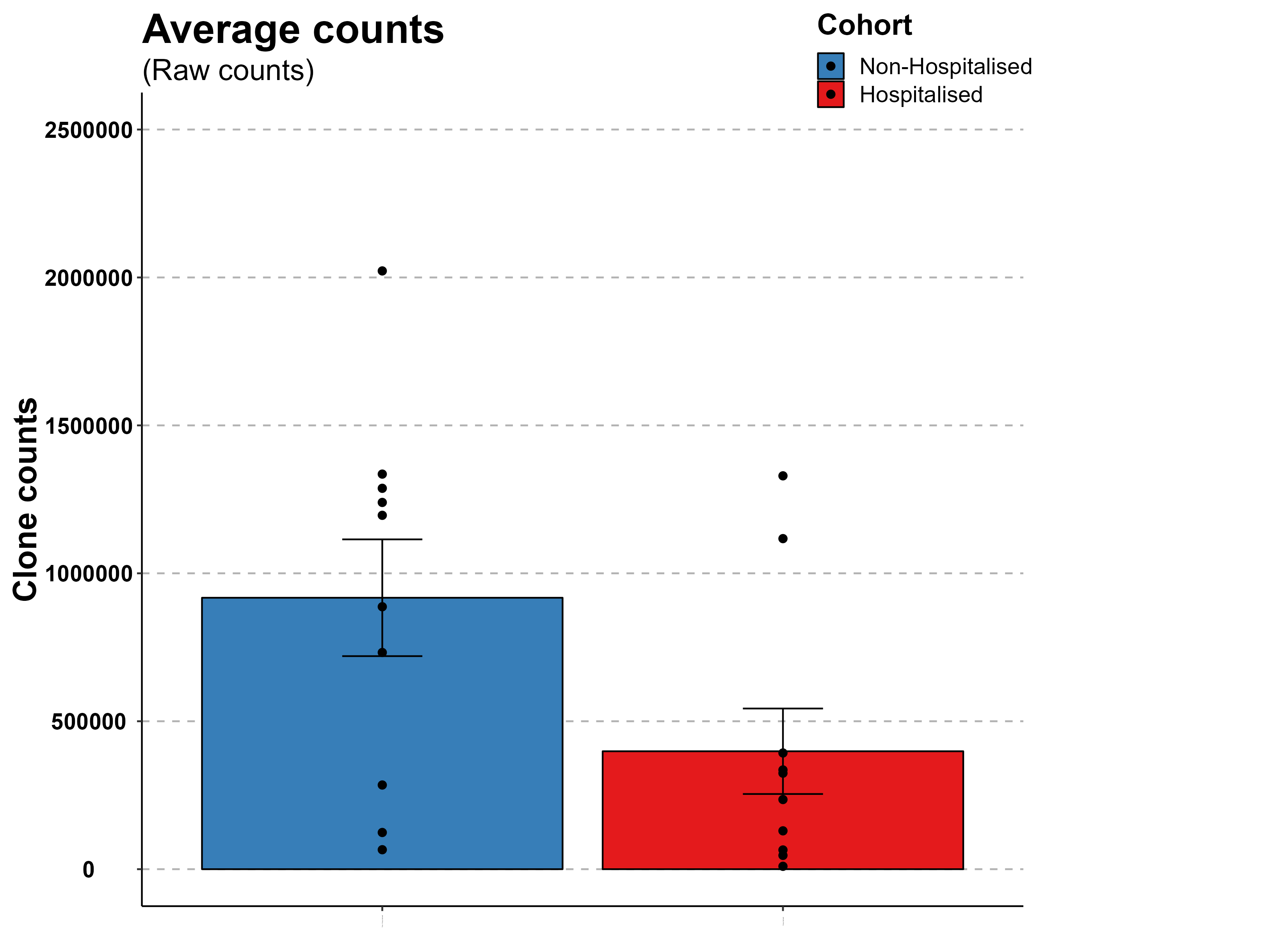

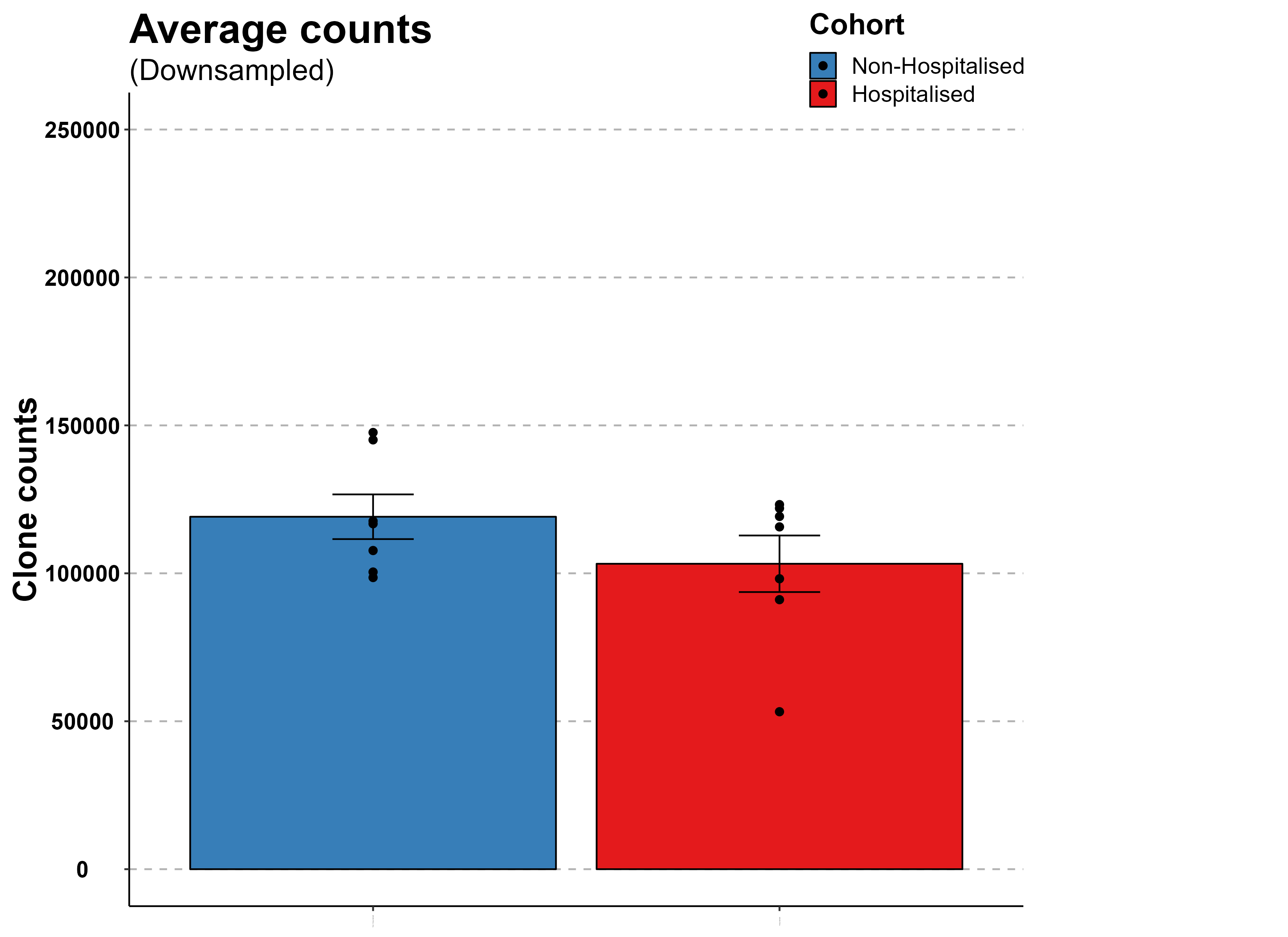


**A**

**B**

**Supplementary Figure 2: Initial repertoire investigation.**
Clone counts for full cohort following MiXCR alignment (Top) and downsampling (bottom) for both TCRα **(A)** and TCRβ **(B)** repertoires

**Supplementary Figure 3:** Location of immunodominant epitopes in SARS CoV-2 genome. Physical structures retrieved from PDB: Nucleocapsid(8FG2), NSP8(7JLT), NSP3(6WUU), pre-fusion spike(7TGW), post-fusion spike(8FDW)


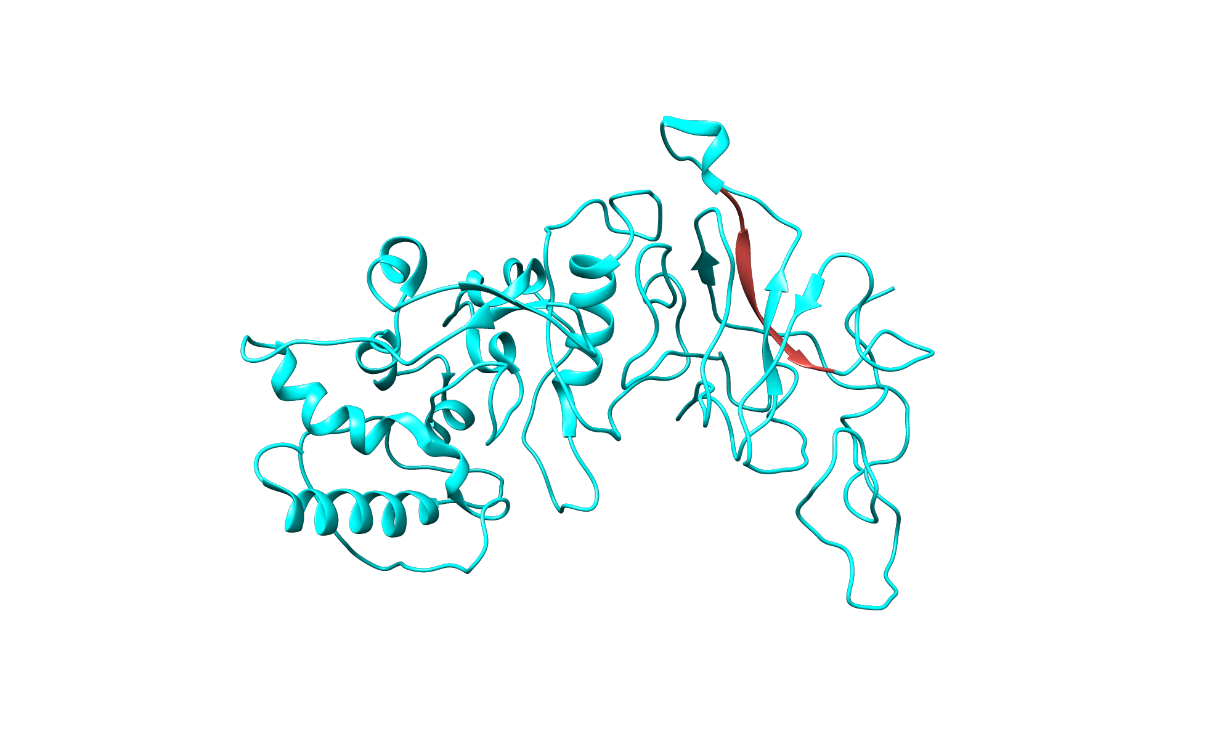

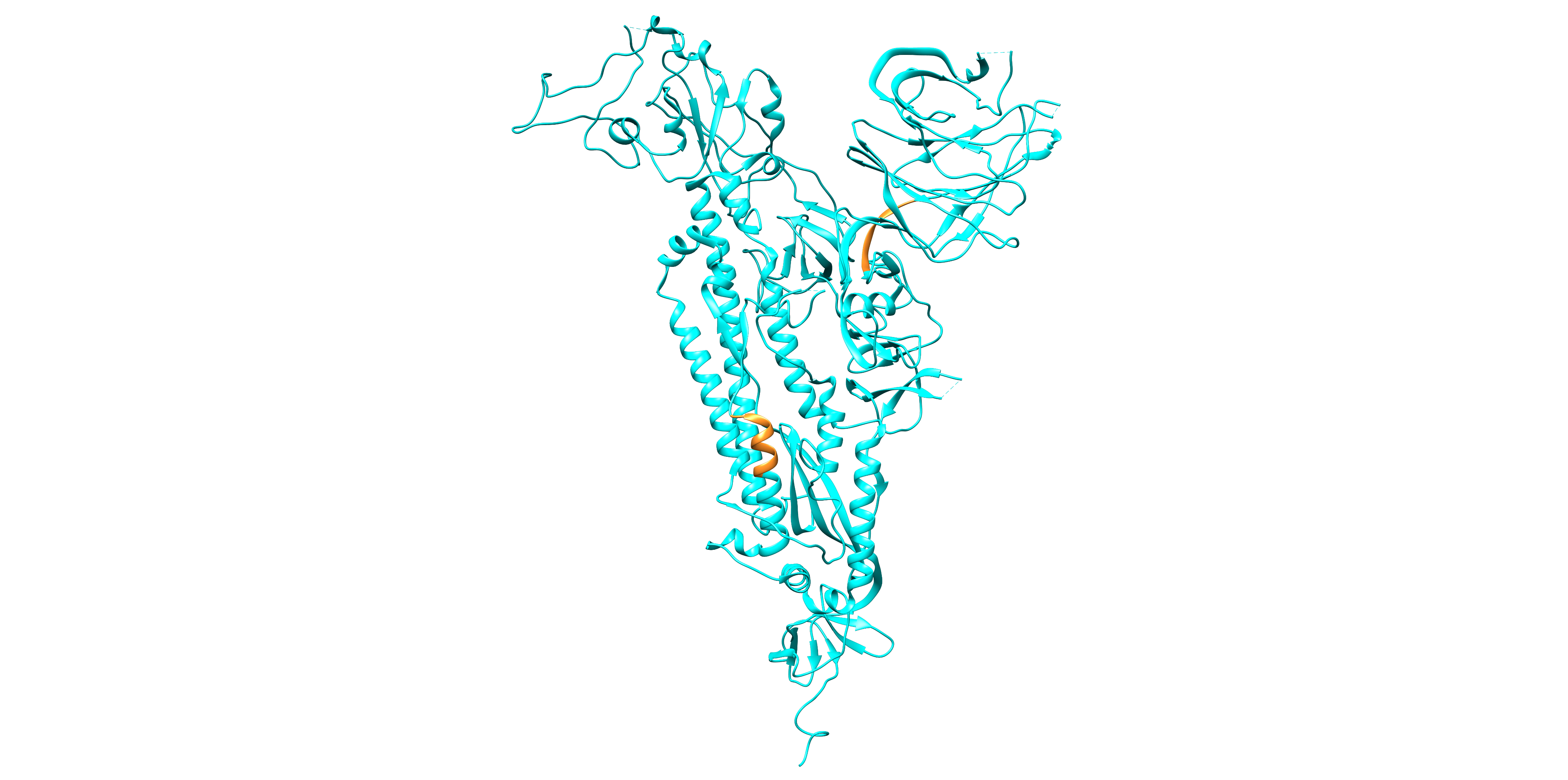


Spike Glycoprotein

Pre-Fusion

LTDEMIAQY

YLQPRTFLL


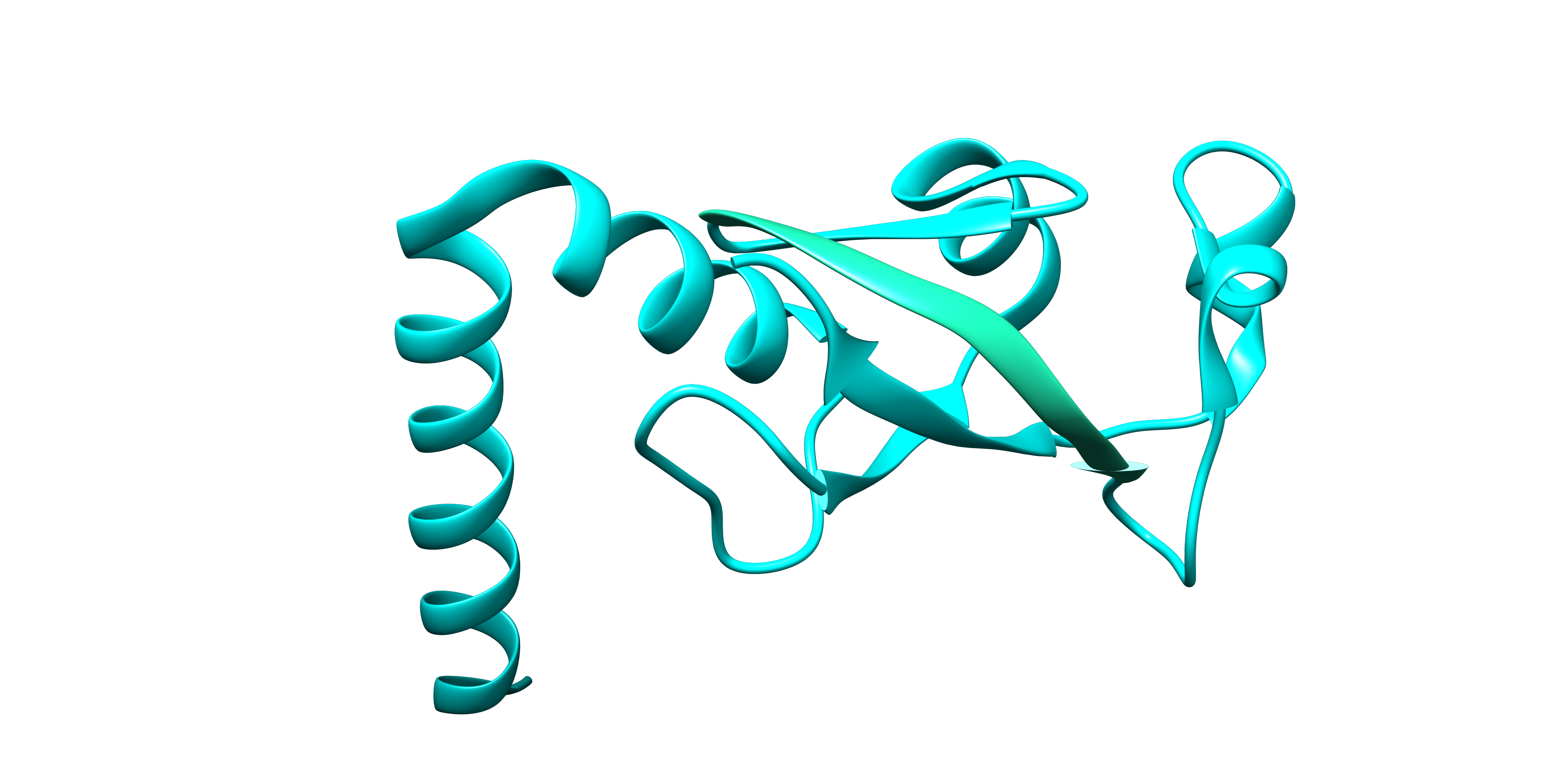


NSP8

ALWEIQQVV

Nucleocapsid

SPRWYFYYL


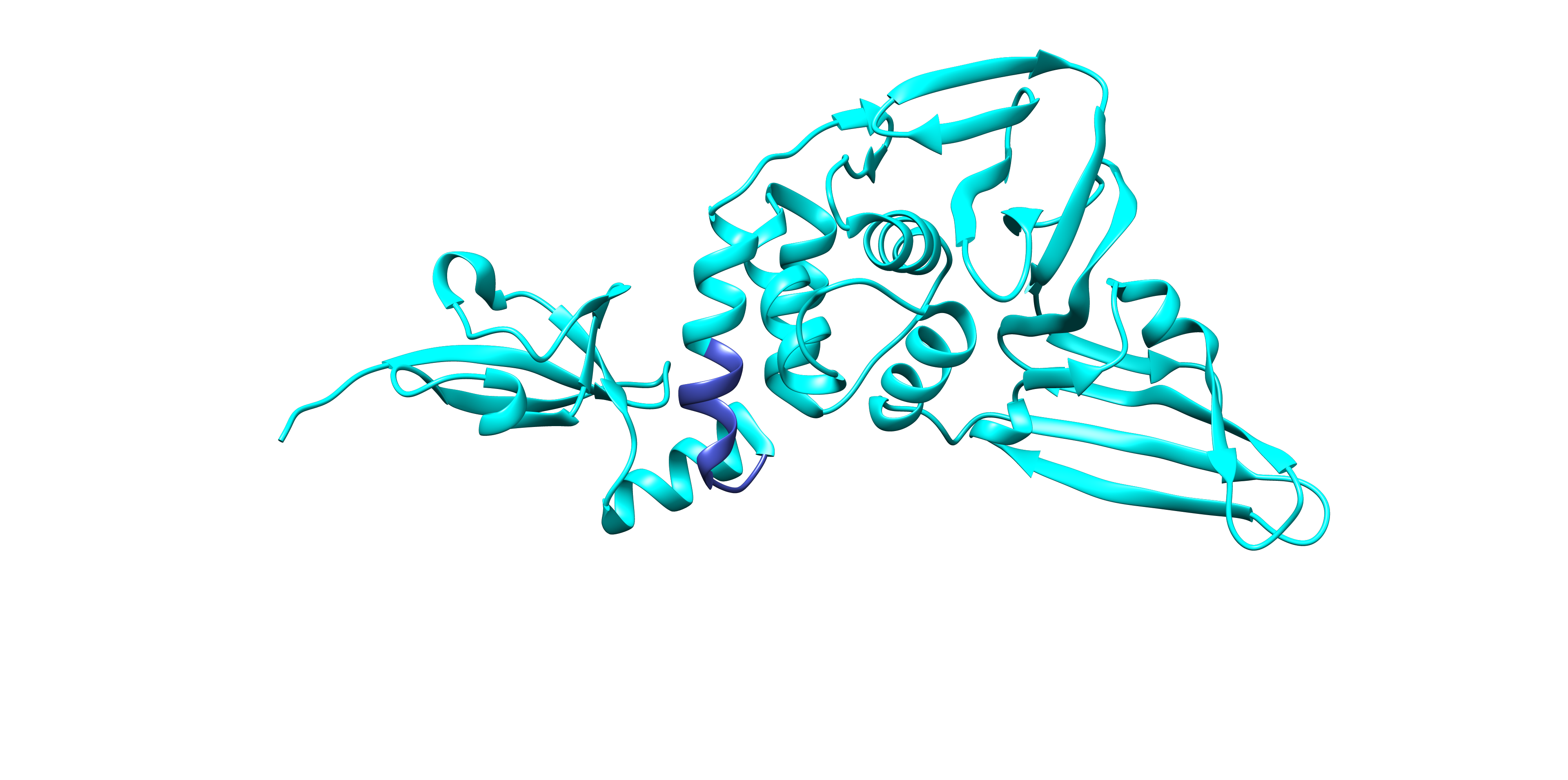


NSP3

TTDPSFLGRY


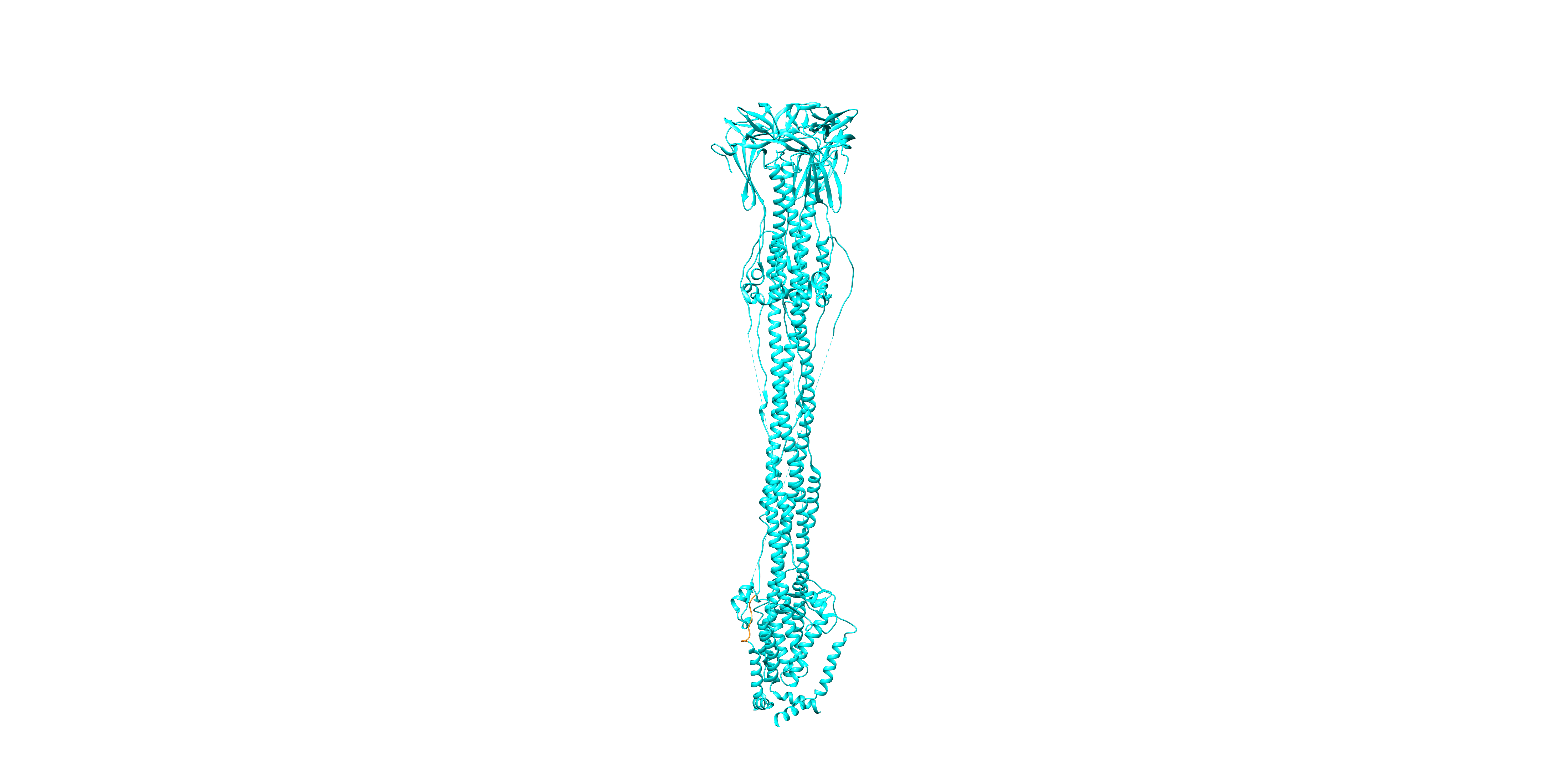


QYIKWPWYI

Spike Glycoprotein

Post-Fusion

**Supplementary Figure 4: (A)** Heatmap of differentially expressed genes between Non-hospitalised and Hospitalised cohorts. **(B)** Volcano plot of differentially expressed genes between Non-hospitalised and Hospitalised cohorts **(C)** Pathway enrichment and directed acyclic graph (DAG) results following gene ontology (GO) enrichment of DEGs **(D)** Relative abundance of HLA genes in Non-Hospitalised and Hospitalised cohorts

**D**

**A**

**B**

**C**


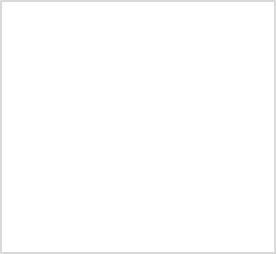

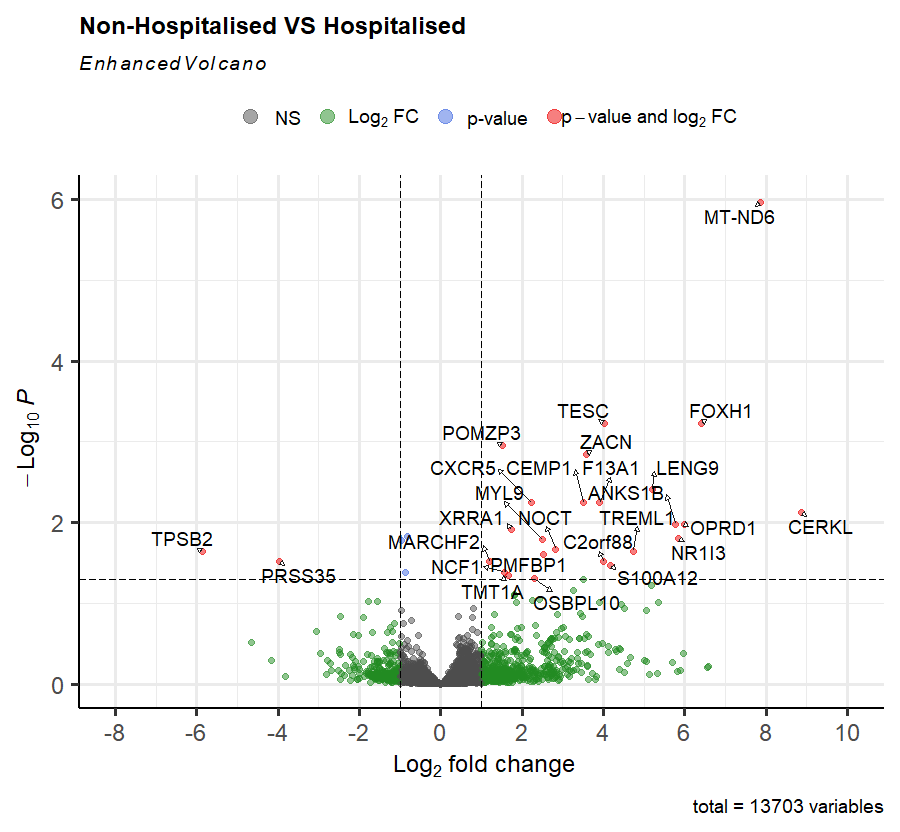

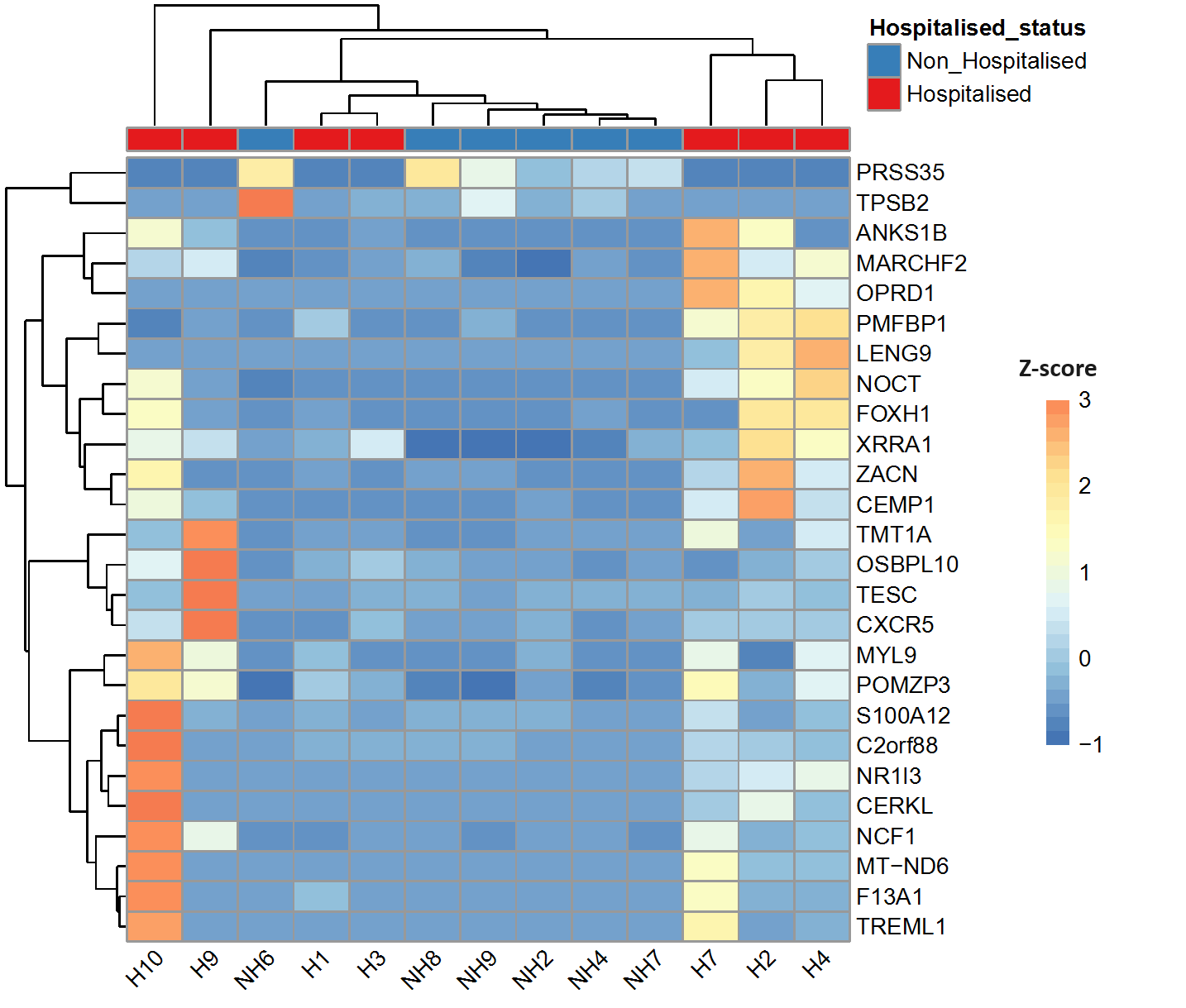

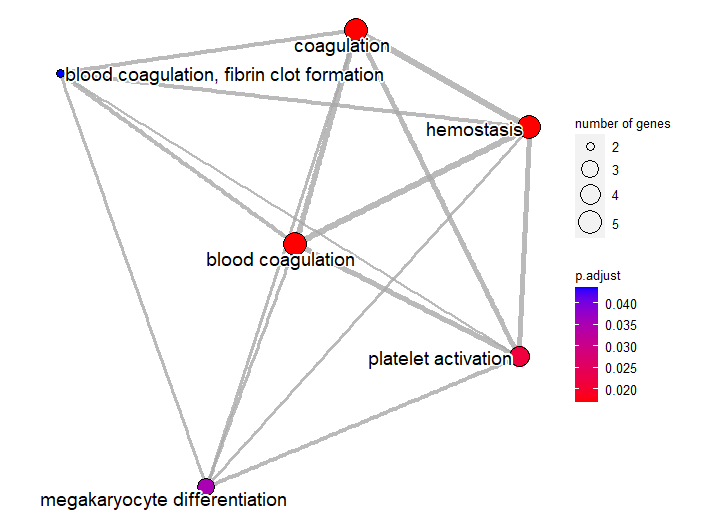

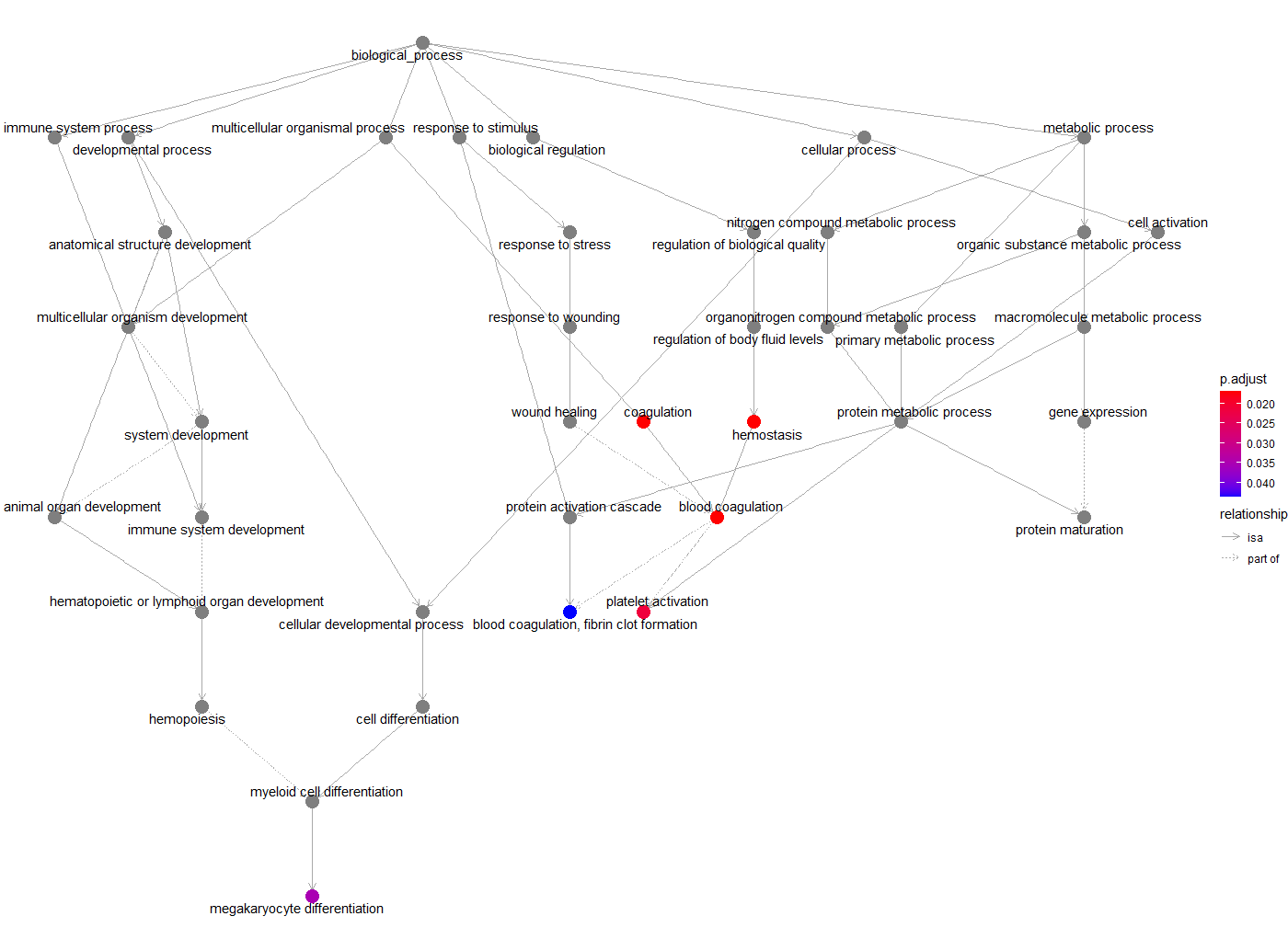

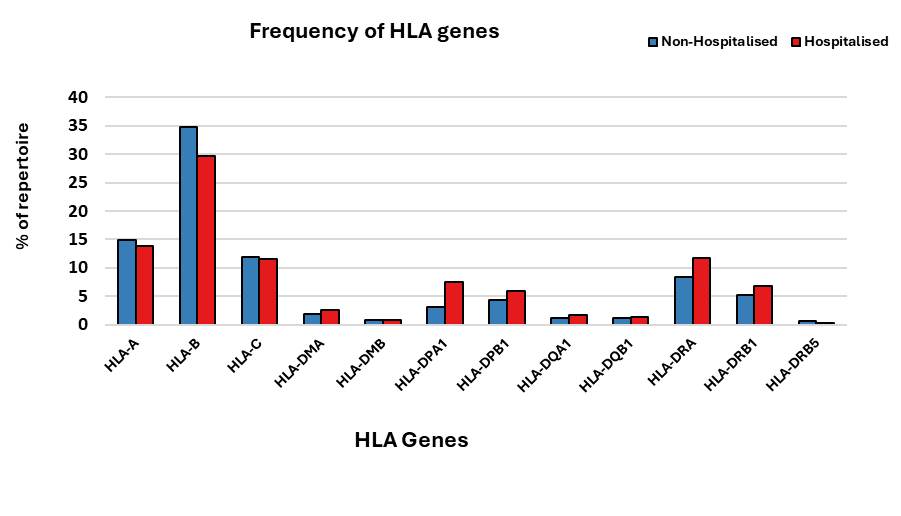

**Supplementary Table 1: Human Leukocyte antigen (HLA) genotyping:** HLA typing and allele frequency was performed on the most commonly occurring HLA genes using arcasHLA for both MHC class I and II alleles. Allele frequency was calculated by using the observed abundance of each gene relative to the total number of mapped reads for each sample. In cases where there were not enough reads to genotype a particular gene a (—) symbol has used.
